# Deciphering the Complexities of Adult Human Steady State and Stress-Induced Hematopoiesis: Progress and Challenges

**DOI:** 10.3390/ijms26020671

**Published:** 2025-01-14

**Authors:** Suzanne M. Watt, Maria G. Roubelakis

**Affiliations:** 1 Stem Cell Research, Nuffield Division of Clinical Laboratory Sciences, Radcliffe Department of Medicine, University of Oxford, Oxford OX3 9BQ, UK; 2 Myeloma Research Laboratory, Adelaide Medical School, Faculty of Health and Medical Sciences, University of Adelaide, North Terrace, Adelaide 5005, Australia; 3 Cancer Program, Precision Medicine Theme, South Australian Health and Medical Research Institute, Adelaide 5001, Australia; 4 Laboratory of Biology, School of Medicine, National and Kapodistrian University of Athens (NKUA), 11527 Athens, Greece; roubel@med.uoa.gr; 5 Cell and Gene Therapy Laboratory, Centre of Basic Research, Biomedical Research Foundation of the Academy of Athens (BRFAA), 11527 Athens, Greece

**Keywords:** human adult hematopoiesis, hematopoietic stem cell, homeostasis, transplantation, regeneration, stress-induced hematopoiesis, lineage tracing, lineage hierarchies, murine models, aging

## Abstract

Human hematopoietic stem cells (HSCs) have traditionally been viewed as self-renewing, multipotent cells with enormous potential in sustaining essential steady state blood and immune cell production throughout life. Indeed, around 86% (10^11^–10^12^) of new cells generated daily in a healthy young human adult are of hematopoietic origin. Therapeutically, human HSCs have contributed to over 1.5 million hematopoietic cell transplants (HCTs) globally, making this the most successful regenerative therapy to date. We will commence this review by briefly highlighting selected key achievements (from 1868 to the end of the 20th century) that have contributed to this accomplishment. Much of our knowledge of hematopoiesis is based on small animal models that, despite their enormous importance, do not always recapitulate human hematopoiesis. Given this, we will critically review the progress and challenges faced in identifying adult human HSCs and tracing their lineage differentiation trajectories, referring to murine studies as needed. Moving forward and given that human hematopoiesis is dynamic and can readily adjust to a variety of stressors, we will then discuss recent research advances contributing to understanding (i) which HSPCs maintain daily steady state human hematopoiesis, (ii) where these are located, and (iii) which mechanisms come into play when homeostatic hematopoiesis switches to stress-induced or emergency hematopoiesis.

## 1. Introduction

Human hematopoietic stem cells (HSCs) have enormous potential and hold the ultimate responsibility for generating billions of blood cells each day, at least during post-natal life. As these cells are critical to survival, and as human research specimens can be difficult to access and analyze functionally in vivo, many studies attempting to define the characteristics and functions of HSCs and their progeny have focused on alternative animal models of hematopoiesis. These have been aided by the more recent introduction of rapidly evolving and sophisticated advanced technologies, such as single-cell sorting, single-cell and spatial multiomics and imaging, single-cell transplantation, cell barcoding, and lineage tracing. Despite these powerful technological advances, these alternative animal models do not always recapitulate the complexity of human hematopoietic stem and progenitor cell (HSPC) hierarchies, lineage commitment, and differentiation patterns under physiological steady state conditions, during ontogeny, and when subjected to hematological or other stressors. Although much more challenging to study, many questions relating to human hematopoiesis are yet to be fully resolved. This review will commence by briefly highlighting some key discoveries or hypotheses (from 1868 until the end of the 20th century), which paved the way for the clinical use of three different sources of human HSPCs for hematopoietic cell transplants (HCTs) and hence for the most successful regenerative therapy to date. This will be followed by a more in-depth review of the challenges faced and progress made in understanding human hematopoiesis, revolving around the definition, characterization, and location of post-natal, and particularly adult, human HSPCs, their contribution to steady state hematopoiesis, and alterations that affect their potentiality and their lineage trajectories as homeostatic hematopoiesis switches to stress-induced or emergency hematopoiesis.

## 2. Background: Identification of Three Sources of Human Hematopoietic Stem and Progenitor Cells for Clinical Hematopoietic Cell Transplants

**Figure 1:**
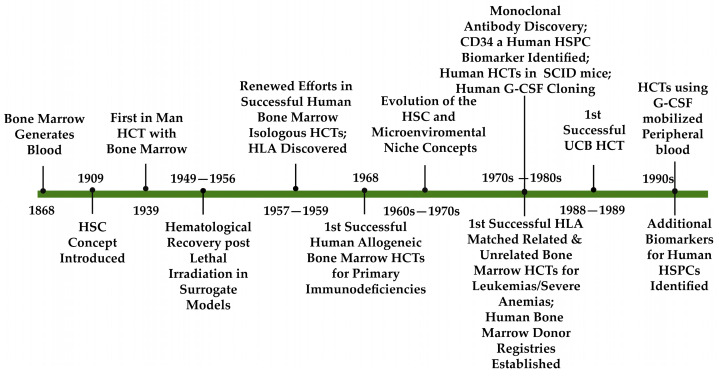
The HSC Concept and Some Notable Human Hematopoietic Cell Transplantation Milestones developed over One and a Half Centuries (from 1868 to 2000). As a brief introduction to the 3 post-natal sources of HSPCs developed for human HCTs by the year 2000, this timeline does not include all studies carried out in different animal models, which have also contributed significantly to our understanding of HCTs and to the evolution of hematological and immunological research as we know it today.

Figure 1 provides an abbreviated diagrammatic historical outline of the pathway to the development of three sources of HSPCs for human HCTs from 1868 to 2000, with some key findings, but by no means all, discussed briefly below. More detailed historical perspectives have been provided elsewhere [1,2,3].

### 2.1. Bone Marrow as the Major Residence for Adult Hematopoietic Stem Cells

Neumann and Bizzozero (with significant contributions from Osler) are credited with identifying, more than a century and a half ago, the bone marrow (BM) as the major site of post-natal mammalian blood formation (reviewed in depth by Cooper [4]). During the late 19th century and early 20th century, such notable investigators as Pappenheim, Dantschkoff, Maximow, and Neumann were strong proponents of the unitarian or monophyletic model of hematopoiesis, which led to the recognition of the HSC as the common multipotent precursor for all blood cells [5,6] However, it was not until 1939 that the first recorded clinical human BM transplant designed to reconstitute hematopoiesis in a patient with aplastic anemia, albeit unsuccessful, was attempted [7].

Renewed research efforts in the late 1940s and 1950s [1,3,8] following the devastation that ensued in the Japanese cities of Hiroshima and Nagasaki with the atomic bomb blasts of 1945 were aimed at preventing or mitigating the adverse effects of radiation-induced damage to the hematopoietic system. Of note has been the recent award of the 2024 Nobel Peace Prize to the Japanese Organization, Nihon Hidankyo, a movement established by Hibakusha or those survivors exposed to the atomic bombings of Hiroshima and Nagasaki, for tirelessly raising awareness of the catastrophic humanitarian consequences of nuclear weapons (www.nobelprize.org/prizes/peace/2024 (accessed on 8 January 2025)). Importantly, early studies in mice demonstrated the radiation-protective effects on hematopoiesis imposed by shielding the murine spleen or femora with lead and the reestablishment of blood cell generation in mice infused with syngeneic BM cells after total body irradiation [9,10]. These and other studies eventually led to the recognition that donor BM cell infusion, rather than humoral factors alone, allowed reconstitution of the irradiated recipient’s hematopoietic and immune systems. Despite such studies, subsequent human BM-derived HCTs reported between 1957 and 1967 were fraught with difficulties and failures (including graft failures, infections, relapse, graft versus host disease (GvHD), and failure of patient long-term survival) [11]. As exemplars, in 1957 in their pioneering contributions to human HCTs, E. Donnall Thomas and colleagues published clinical studies, in which six patients received intravenous normal isogenic donor BM infusions following high-dose total body irradiation; however, only one patient transiently engrafted [12] (reviewed in [2]). Then, in 1959, hematological reconstitution was reported in two siblings with advanced leukemia, who received identical twin BM transplants after high-dose total body irradiation; however, both patients relapsed with the leukemia within approximately 6 months of the HCT [13] (reviewed in [2]). Subsequently, E. Donnall Thomas’ persistence in leading research efforts in this area of hematology was recognized by the award of the 1990 Nobel Prize for Physiology or Medicine for his outstanding contribution to the development of bone marrow transplantation (www.nobelprize.org/prizes/medicine/1990 (accessed on 8 January 2025)).

Of further importance was the research that paved the way for the discovery of the major histocompatibility complex (MHC) in animal models (some key examples include [14,15,16,17,18,19,20]). Notably, three further publications in 1958 by Dausset, van Rood, and Payne and colleagues [21,22,23] laid the foundations for the in-depth description of the human leukocyte antigen (HLA) complex and its importance in histocompatibility matching between human allogeneic HCT donors and recipients to aid engraftment, to lessen GvHD, and to promote graft versus leukemia (GvL) effects [24]. For “their discoveries concerning genetically determined structures on the cell surface that regulate immunological reactions”, Snell, Benacerraf, and Dausset received the 1980 Nobel Prize in Physiology or Medicine (www.nobelprize.org/prizes/medicine/1980 (accessed on 8 January 2025)). More comprehensive reviews of early research in the histocompatibility arena, as well as further pre-clinical studies in rodents and larger animal models, are described in more detail elsewhere [3,24,25]. These led to a return to clinical studies, which eventually improved success rates for human allogeneic HCTs [26]. Importantly, in 1968, the first successful human allogeneic BM transplants in patients with primary immunodeficiency disorders were published by three groups [27,28,29]. Such studies established the human BM as the main site of hematopoietic cell residence.

### 2.2. The Concept of the Hematopoietic Stem Cell Niche and the Search for an In Vivo Human Hematopoietic Stem Cell Assay

The concept of specialist microenvironmental niches, intimately involved in regulating HSPC behavior in vivo, was further developed in the 1960s and 1970s, evolving from much earlier studies or observations, including those described in 1941 by Röhlich [30] (reviewed in [31,32,33,34]). These advances were complemented by efforts to identify and quantitate HSPCs. Of particular note were seminal murine studies in the early 1960s, which led Till, McCulloch, and colleagues [35,36,37,38] to establish the spleen-colony-forming unit (CFU-s) assay. This was characterized by the formation in mice of macroscopic spleen colonies 1–2 weeks after syngeneic BM transplantation into irradiated mice. A proportion of these CFU-s was reported to possess short-term “self-renewal” ability and the capability of differentiating into erythromyeloid cells. Based on studies conducted between 1964 and 1967, Curry, Trentin, and Wolf proposed the existence of specific hematopoietic inductive microenvironments (HIMs) in the spleen; these promoted erythroid differentiation from HSCs and differed from those in the BM that promoted granulopoiesis [39,40,41]. This concept of the niche was further developed by Scholfield [42]. More robust technologies (including cell barcoding, flow sorting and analyses, and transplantation of enriched murine bone marrow HSPC subpopulations) were subsequently used to characterize CFU-s subsets in more detail. These conclusively demonstrated that a proportion of late-appearing (day 13.5) spleen colonies were derived from multilineage CFU-s with the capability of generating erythroid, myeloid, megakaryocytic, and B lymphoid cell lineages, although the predominant cells in the colonies were erythroid [43]. These CFU-s assays formed the basis for the later functional identification of murine and human putative HSCs in serial long-term murine transplant models. The in vivo CFU-s assays were complemented by the development of in vitro clonogenic assays, with the originating cell known as the colony-forming cell or CFC. These assays were developed for murine hematopoietic progenitor cells (HPCs) and subsequently adapted for human HPCs; they measure the differentiation capacity of clonogenic HPCs or CFCs when plated into semi-solid media with appropriate growth factors and have played a key role in defining hematopoietic lineage trajectories [44,45,46,47,48,49,50,51,52]. Other longer-term single-cell in vitro assays, such as the cobblestone-area-forming cell (CAFC) and long-term-culture-initiating cell (LTCIC) assays [53,54,55,56,57,58] have relied on the growth on competent stromal cell layers of the more immature murine or human HPCs (clonogenic or at limiting dilution), which, when serially replated (as individual colonies or paired daughter cells into CFC or lymphoid-defined conditions) have allowed the quantitation of multipotent or more restricted erythromyeloid and/or lymphoid precursors, which lacked or possessed limited “self-renewal” potential [59,60,61]. Competent stromal cell lines, such as MS-5, OP9, and OP9-DL1 with the respective abilities to support clonogenic human B and myeloid cell differentiation and lympho-myeloid and T cell differentiation, are commonly used in these CAFC or LTCIC assays [62,63,64,65,66]. Stromal-free or stromal-based cultures have been used with index sorting and single-cell (sc)RNA-seq experiments to assess human HSPC lineage trajectories or branching points [67,68].

A step forward, which led to the development of murine xenograft models to assay human HSPCs with defined repopulating and/or self-renewal capabilities, occurred with the 1988 description of engraftment in vivo of human lymphoid and/or myeloid cells in severe combined immunodeficient (SCID) mice, often referred to as SCID repopulating cells (SRCs) or repopulating HSCs [69,70,71]. Other notable studies, encompassing the transplantation of donor human HSPCs into larger animal models, included the pre-immune fetal sheep transplant model [72]. Alternatively, human HCTs were compared with non-human primate HCTs, where hematopoiesis most closely resembles that in humans [73].

### 2.3. Further Advances in Human Hematopoietic Cell Transplants and in Identifying Biomarkers for Human Hematopoietic Stem and Progenitor Cells

Current routine clinical HCTs are designed to replace the recipients’ compromised hematopoietic system totally or partially with functionally normal HSCs and their progeny. These HCTs may be autologous or allogeneic, with HSCs principally sourced from the BM, mobilized peripheral blood (mPB), or umbilical cord blood (UCB) of the respective patient or appropriate human donor(s), depending on clinical indications and treatment strategies [74]. Historically, the 1970s and 1980s saw the first successful human HCTs resulting in long-term survival for some patients with leukemias and severe anemias (mostly from matched sibling donors), the establishment of the first BM donor registries, and the first unrelated HLA-matched HCTs and HLA-identical sibling UCB HCTs [2,3,75,76]. This coincided with the development of fluorescence-activated cell sorting [77], the discovery of CD34 as an HSPC biomarker [78], the development of new isolation methods for human CD34+ HSPCs [79] and advances in the identification of hematopoietic growth factors [80] with, importantly, the cloning of human granulocyte-colony stimulating factor (G-CSF) [81] (reviewed in [82,83]). In the late 1980s and 1990s, G-CSF-mobilized peripheral blood (PB) gradually became established as a source of HSCs for HCTs [83] and additional biomarkers (e.g., CD90, CD133, CD164) were identified on human HSPCs [84,85,86,87,88].

Although commenced in the 1980s [89,90], HLA-haploidentical grafts sourced from family members showed poor survival outcomes following HCTs until post-transplant cyclophosphamide was introduced for the prevention of GvH disease (reviewed in [91,92]). Even though increased use of UCB as a cell source for HCTs was observed during this millennium, improvements in HLA-haploidentical HCTs [91,92,93,94] have nevertheless led to a more recent expansion of the donor pool, a decline in UCB HCTs [95], and a preference for mobilized peripheral blood, as opposed to BM [93]. Given that donor age is considered of major importance for human allogeneic HCT outcomes (reviewed in [93]), Shi and colleagues [95] have argued in favor of maintaining readily available, HLA-typed, and banked UCB units for HCTs as this source of HSPCs, amongst other considerations, should be exposed to less replicative stress and hence better maintain their genome integrity.

### 2.4. Global Clinical Hematopoietic Cell Transplants Now Exceed 1.5 Million

Current HCTs offer, or contribute to, potential curative therapies for certain hematological malignancies (e.g., leukemias, lymphoproliferative disorders), some solid tumors, and specific non-malignant conditions (including BM failures, immune deficiencies, hemoglobinopathies, inherited metabolic diseases, autoimmune diseases) [74,96,97]. An estimated one million HCTs were carried out worldwide from 1957 to 2012 [98], making this the most successful regenerative therapy to date. By 2019, just prior to the negative impact of the Coronavirus disease 2019 (COVID-19) pandemic (caused by severe acute respiratory syndrome coronavirus 2 or SARS-CoV-2) on HCT activity, total HCTs were estimated by the Worldwide Network of Blood and Marrow Transplantation (WBMT; www.wbmt.org) to be around 1.5 million [96]. These HCTs comprised slightly higher numbers of related (53.6%) than unrelated transplants and of autologous (53.5%) compared to allogeneic transplants [96]. In Europe, HCT activity decreased considerably in 2020, improved in 2021, and then decreased again in 2022 for both autologous and allogeneic transplants and for most indications [97]. This decline was associated with the recent SARS-CoV-2 pandemic but may also reflect the availability of new therapeutic options for patients eligible for HCTs [97].

With significant technological advances during the 21st century, efforts are being made to improve the precision or personalization of HCTs to meet specific patient needs and demographics to enhance outcomes. These include stratifying patients based on their disease and clinical status and incorporating the best available donor selection, graft type, conditioning, novel targeted cellular and molecular therapies, and post-HCT maintenance [93,97]. Importantly, the complexity, reliability, safety, and success of HCTs have been highly dependent on major scientific and clinical breakthroughs in hematology and immunology, on the availability of appropriate infrastructure and technological advances, and on the significant input and expertise of highly experienced medical and scientific specialists from various fields [74,96,97]. Furthermore, historical transplant studies have led investigators to examine the dynamics and mechanisms of regenerative human hematopoiesis post-transplantation and during other significant stresses more closely and to analyze how these differ from those that define physiological steady state hematopoiesis in healthy individuals.

## 3. Progress and Challenges in Defining Adult Human Hematopoietic Stem and Progenitor Cells in Steady State and Stress-Induced Hematopoiesis

### 3.1. Daily Turnover of Hematopoietic Cells Under Steady State Conditions as a Reflection of Adult Human Hematopoietic Stem Cell Potential

**Figure 2:**
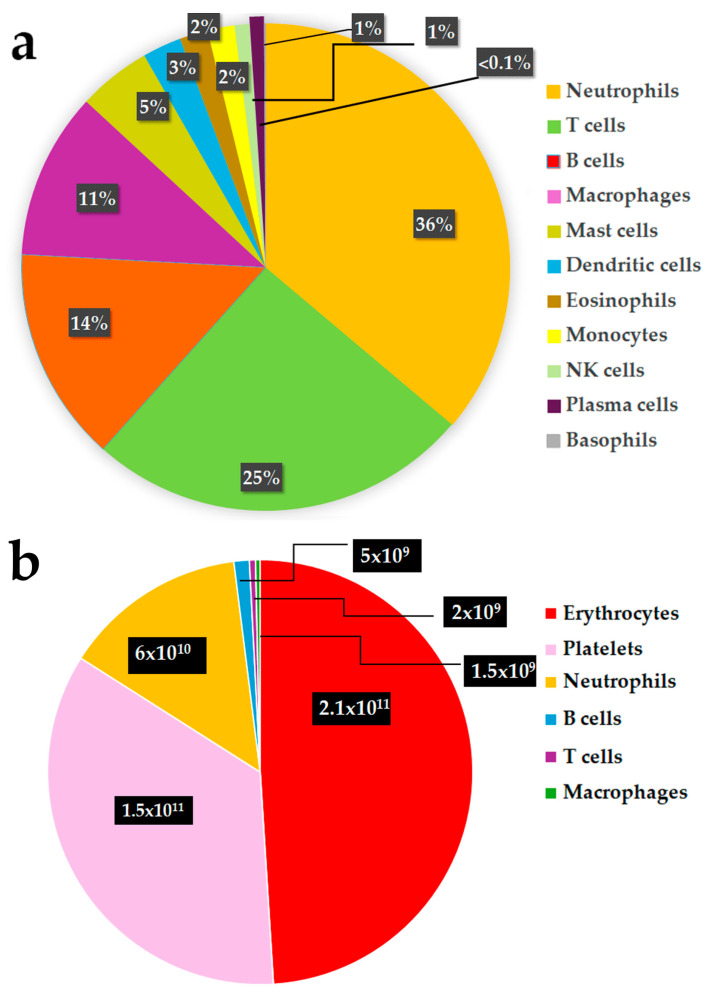
Distribution and Turnover of Human Hematopoietic Cells. (**a**) The relative proportions of eleven nucleated immune cell subsets distributed throughout the human body of an average healthy young adult male. (**b**) Percent daily turnover rate of six mature hematopoietic cell types in an average healthy young adult male. Note the high daily turnover rates of erythrocytes, platelets, and neutrophils. These calculations vary with age, gender, and stage of ontogeny and are adapted from the data in [99,101,103].

Human HSCs have traditionally been considered to be ultimately responsible for sustaining most blood and immune cell production throughout adult life. The enormous potential of human HSCs and their more committed progenitor cells is reflected in the extremely high daily turnover and regeneration of mature blood cells under physiological or homeostatic conditions. Indeed, it has recently been estimated that c. 86% of the new cells generated daily in a healthy young human adult are of hematopoietic origin [99]. This equates to the daily generation of c. 10^11^ to 10^12^ hematopoietic cells, with the vast majority comprising relatively short-lived platelets, with important roles in hemostasis and in the innate and adaptive immune systems, and erythrocytes, the low-oxygen sensors and oxygen transporters (Figure 2a) [99,100,101,102]. In contrast, neutrophils, a first line of immune defense against pathogens, are estimated to have the highest daily turnover rate relative to other nucleated white blood and immune cells, with this being significantly higher than that of lymphoid cells and other myeloid cell types [103] as illustrated in Figure 2b. Thus, the most replenished mature hematopoietic cells under steady state conditions are those that do not divide but are critical to life.

To understand the basis for this daily turnover of these and other mature hematopoietic cells in the adult human, it is important to determine (i) which hematopoietic stem or progenitor cell populations maintain this steady state hematopoiesis on a daily basis, (ii) where these are located, and (iii) which mechanisms come into play when homeostatic hematopoiesis switches to stress-induced or emergency hematopoiesis.

### 3.2. Defining Post-Natal Human Hematopoietic Stem Cell Populations

#### 3.2.1. The Hallmarks of Stemness: Not All Human Hematopoietic Stem Cells Are Created Equal

Given their capacity to generate and maintain lifelong hematopoiesis, it has been suggested that, at the population level, post-natal HSCs in vertebrates, like other stem cells, exhibit six combined or complementary hallmarks of stemness, viz., *(1) self-renewal and longevity, (2) multipotency, (3) transplant ability, (4) plasticity, (5) dependence on niche signals, and (6) maintenance of genome integrity* [104]. These characteristics have generally been useful when identifying and isolating human functional HSCs and segregating them from their progeny, although, as pointed out by Breumer and Clevers [104], enriched HSCs are heterogeneous and may be lineage biased or primed. Understanding this heterogeneity in human HSCs and their more differentiated HPC offspring has relied heavily on the differential distribution of specific surface biomarkers to enrich cell subsets, the determination of their potency and their differentiation trajectories in vitro and in vivo, and their single-cell molecular, cellular, and phenotypic profiles [104,105,106,107]. However, an important challenge has been to ascertain if the latter profiles and their functions are affected by the prior manipulation of these cells outside their natural microenvironment and if they reflect and are consistent with physiological steady state or stress-induced hematopoiesis.

#### 3.2.2. The Challenges of Researching Human Hematopoiesis

Efforts by many investigators, especially over the past 4–5 decades, to define and purify human HSCs and to characterize their pathways of commitment and differentiation for each blood and immune cell lineage generated during different stages of ontogeny were boosted by the Nobel-Prize-winning description of the hybridoma technique for generating monoclonal antibodies (Mabs) [108]. Historically, this approach initially highlighted two key cell surface biomarkers, CD34 and CD133, expressed on, but not restricted to, human HSCs or HSPC subsets, including those with the ability to robustly and durably reconstitute multilineage lympho-myeloid hematopoiesis in human transplant recipients over the median to long term. These studies supported the premise that HSCs are present in the CD34+ and/or CD133+ cell compartments and served as an indication of their longevity, multipotency, and transplant ability. Indeed, effective long-term outcomes have been reported in a proportion of patients receiving human allogeneic or autologous CD34 and CD133 enriched HCTs [109,110,111] and, despite some significant challenges, in certain patients receiving autologous HCTs of ex vivo genetically engineered CD34+ HSPCs to treat such monogenic blood disorders as primary immunodeficiencies, hemoglobinopathies, BM failure syndromes, or inherited neurometabolic disorders (see recent reviews [112,113,114]). Although not all patients undergoing such gene therapies demonstrate sustained and optimal hematopoietic outcomes over the medium to long term [115,116,117], lentiviral-mediated gene engineering of autologous CD34+ HSPCs coupled with myeloablative conditioning prior to transplant has provided more consistent and enhanced levels of patient engraftment than γ-retroviral-mediated gene transfer approaches (reviewed in [112,113,114,118]) with stable and polyclonal hematopoietic reconstitution (commencing from about 1–2 years post-HCT) described in a series of patients followed for up to a decade or more (reviewed in [112,114,116,119,120,121,122,123]). Since these therapeutic strategies were first introduced, it has become clear that human HSCs constitute only rare subsets within the heterogeneous CD34+ or CD133+ HSPC pool. Clonal tracking via longitudinal genomic integration site analyses of hematopoietic cells derived from engrafted genetically engineered autologous human BM or mPB CD34+ HSPCs have, for example, demonstrated long-term persistence not only of multipotent HSCs and multipotent progenitors (MPPs) but also of lymphoid- or myeloid-biased clones [115,123]. Novel genome editing combined with artificial intelligence approaches is expected to provide improved patient outcomes, as well as better insight into human HSC heterogeneity in terms of function and potency, undoubtedly deepening our future knowledge of human hematopoiesis [124,125]. While the first human therapy based on CRISPR-Cas9 editing for severe hemoglobinopathies received regulatory approval in the UK and USA towards the end of 2023 (reviewed in [126]), these newer strategies still face significant challenges in their translation from bench to bedside and await long-term follow-up [125,126,127,128,129].

Since human HSCs were identified in rare subsets of CD34+ or CD133+ HSPCs, dissecting the heterogeneity of the human HSPC compartment, until recently, relied on the differential expression profiles of a limited number of additional cell surface biomarkers. The next landmark was the introduction of single-cell transcriptomics profiling and then of advanced high-resolution single-cell multiomics approaches, complemented by functional in vitro clonal and durable in vivo transplantation assays in surrogate animal models, primarily in immunodeficient mice [130]. It is well established that the outcomes of such in vivo surrogate transplant assays can, however, vary with the human HSPC source and the methodology for isolating or enriching HSPC subsets [79,131,132,133,134,135,136], ex vivo manipulation of the human HSPCs [137,138,139,140], the mouse strain used [132,140,141,142], the murine conditioning regime [127,140,143], the human donor cell number transplanted, the use of feeder cells with the transplant, the route of transplantation, the murine BM microenvironment, whether primary or serial transplants are assessed and at which point in time this occurs, the relative level of engraftment and lineage read-out of human donor HSPCs, and other human donor and murine recipient characteristics ([79,131,136,144,145,146] and references therein). This lack of methodological standardization amongst research teams often makes comparisons of different human HSC purification strategies difficult.

Additionally, because of access issues, many studies have used circulating HSPCs from human UCB or mPB in preference to the resident HSPCs of human BM and to HSPCs in fetal tissues. However, as the functions and characteristics of circulating HSPCs can differ from BM-resident HSPCs, adding to the complexity in understanding the dynamics of physiological steady state and stress-induced hematopoiesis during human post-natal life, there have been renewed efforts recently to understand similarities and differences in lineage programs of human HSPC subsets derived from these different sources and at different stages of ontogeny [147,148,149,150,151,152].

### 3.3. Enriching Post-Natal Human Hematopoietic Stem Cells Using Limited Sets of Biomarkers

#### 3.3.1. CD34 as a Key Human Hematopoietic Stem and Progenitor Cell Biomarker

Various studies have indicated that cell surface sialomucin, CD34, is present on a mean of ≤1 to over 5% of fetal blood and fetal liver mononuclear cells and post-natally sourced human UCB, BM, and mobilized and non-mobilized or steady state PB mononuclear cells, with the frequency dependent on HSPC source [153]. As indicated above, the human CD34+ HSPC compartment in these tissues is composed mostly of lineage-restricted HPCs and much rarer long-term repopulating HSCs. Notably and based on earlier hematopoietic research (reviewed in [79,131,153,154,155]), John Dick’s group contributed significantly to one of the most common enrichment strategies for human post-natal CD34+ HSCs. They demonstrated that long-term multilineage lympho-myeloid engraftment activity was contained within both CD90- and CD90+ subsets of 9Lin-CD34+CD38-CD45RA- human UCB HSPCs, when assessed by serial transplantation (after 20+12 to 14 weeks, respectively) into sublethally irradiated female NSG mice [156]. These cell subsets could be further enriched for HSCs based on CD49f (integrin α6) positivity, with c. 9.5% (1 in 10.5) of 9Lin-CD34+CD38-CD45RA-CD90+CD49f+ and c. 4.5% (1 in 22.1) of 9Lin-CD34+CD38-CD45RA-CD90-CD49f+ cells displaying multilineage lympho-myeloid engraftment 20 weeks after transplantation at limiting dilution into sublethally irradiated NSG mice. The ability of a proportion of single 9Lin-CD34+CD38-CD45RA-CD90+CD49f+ human UCB cells, further fractionated for high Rhodamine 123 efflux (the Rho^lo^ subset), to serially repopulate NSG mice provided evidence for the self-renewal capacity of individual human UCB HSCs. Furthermore, loss of CD49f from the 9Lin-CD34+CD38-CD45RA-CD90-CD49f+ HSPC subset defined short-term (ST-)HSCs or MPPs, characterized as having the ability to transiently repopulate both lymphoid and myeloid lineages [156].

#### 3.3.2. CD133 Marks Primitive CD34- and CD34+ Human Hematopoietic Stem Cells

Human CD133 (also known as prominin-1) has also been used as a key cell surface molecule for identifying and enriching human HSPCs [157,158], (reviewed in [136,159,160,161]). The CD133 molecule is a penta-span and cholesterol-binding membrane glycoprotein containing three intracellular domains (IC1-3) and three extracellular domains (EC1-3), with five potential N-glycosylation sites located on the EC2 domain and four on the EC3 domain (reviewed in [159,160]). It is one of two prominin genes (*PROM-1* and *PROM-2*) identified in humans, with the *PROM-1* gene possessing six known alternative promoters [161]. Although the full-length human CD133 protein comprises 865 amino acids, human CD133 may exist as multiple transcripts and differential splice and post-translationally modified variants in different tissues and cell types [158,159]. Early studies, for example, indicated that the AC133 Mab, often used to identify CD133 on the extracellular surface of human HSPCs, could bind CD133 isoforms 1 and 2 [86,160,161]. Further evidence demonstrated that post-translational N-linked glycosylation contributes to the identification of the AC133-defined CD133 epitope on the cell surface of human HSPCs, possibly by affecting the stability, folding, conformation, and trafficking to and/or regulatory role at the cell membrane of the CD133 molecule [160,161] (reviewed in detail in [159]).

The AC133-defined CD133 glycoprotein has been detected on the surface of human CD34^bright^ HSPC subpopulations from fetal liver, fetal and adult BM, UCB, healthy steady state adult PB, and adult mPB [86,149,157,160,161,162,163,164,165,166,167]. Interestingly, a recent report, using spatial and single-cell transcriptomics, suggests that the AC133-defined cell surface epitope of CD133 is first acquired as human nascent HSCs transition to the fetal liver during human embryonic development and begin to mature towards a post-natal, homeostatic HSC state [167]. These researchers propose, and support the recently held view, that immature HSCs (or pre-HSCs), which initially lack transplant potential in surrogate models, emerge predominantly from the aorta–gonad–mesonephros (AGM) region of the human embryo between CS14 to CS17 by the process of endothelial to hematopoietic transition (EHT) before traveling to and colonizing human fetal liver around Carnegie Stage (CS)17, where they subsequently undergo limited expansion and mature into transplantable HSCs, while gradually acquiring multipotency and becoming predominantly quiescent (reviewed in [152]). This maturation process requires an enhanced MLLT3-regulated HSC self-renewal program, suppression of fetal *LIN28B*, *IGFBP2*, and posterior *HOXB* gene programs, and acquisition of cell surface AC133-defined CD133 and HLA-DR biomarkers (reviewed in [152,167]). Where assessed in xenotransplants, human HSPCs expressing the AC133-defined CD133+ surface epitope reconstitute hematopoiesis, although post-transplant assessment times have varied substantially amongst different surrogate models [86,136,137,140,165].

Despite the overwhelming characterization of CD34+ selected cells, rare cell surface CD34-negative (CD34-) repopulating HSCs have been identified post-natally in the Lin-CD38- subset of human UCB, adult BM, and mPB [72,86,133,157,168,169,170,171,172]. They are generally considered to be more primitive than CD34+ HSCs, express the AC133-defined CD133+ surface biomarker, along with such other biomarkers as CD164 or CD93, and have been positioned at the apex of the human HSC hierarchy ([85,133,157,170,171,172] and references therein). In contrast to CD34, the AC-133-defined CD133 epitope has been found to selectively localize to plasma membrane protrusions of more primitive human HSPCs that are cell surface positive or negative for CD34 [133,173,174].

While cell surface CD34-negative HSCs represent a small fraction of CD133+ cells (<1–2% in adult human BM and UCB), three independent studies indicate that 35–83%, 36–70%, and 59–89% of human UCB, adult BM, and mPB CD34+ cells, respectively, are cell surface AC133/CD133+ [170,171,172] (reviewed in [159]). Interestingly, significantly fewer CD34+ cells in healthy steady state adult human PB cells expressed cell surface CD133 when compared to CD34+ cells in UCB and mPB [169,170,171,172]. A similar phenomenon has been observed in the BM of children with sickle cell disease and β-thalassemia, where the CD133-CD34+ cells comprised mostly B lymphoid progenitor cells which do not show long-term engraftment in murine xenograft assays [127,129]. Significant enrichment of repopulating human UCB CD34- HSCs to approximate frequencies of one in eight cells has been achieved using CD133 positivity combined with the differential expression of other cell surface markers, for example the 18-lineage-negative (18Lin-) CD34-CD38-CD133+GPI-80+ cell subset [170,171]. GPI-80 had previously been detected by another group on self-renewing and engrafting human fetal liver HSPCs [175]. Intra-tibial transplantation of limited numbers of these human UCB 18Lin-CD34-CD38-CD133+GPI-80+ cells compared to the 18Lin-CD34+CD133+GPI-80+ subset into sublethally irradiated NOG or NSG mice has revealed similar human multilineage lympho-myeloid cell reconstitution patterns over a 50-week period [170,171]. Primary single-cell transplants of these two cell subsets were also assessed by secondary and tertiary transplantation (at up to 22-week intervals) in NSG mice, with results suggesting that both subsets possessed extensive self-renewal ability [136,170]. Notably, although these CD34+ and CD34- subpopulations demonstrate heterogeneity at the single-cell gene expression level, their gene expression patterns are distinct from one another [171,176]. At the cell surface biomarker level, both CD34+ and CD34- repopulating subsets expressed higher levels of CD133, as well as higher levels of CD201 and CD49f, than were detected on non-repopulating HSPCs [170]. Sumide et al. [170,171] further reported that CD133+CD34- HSCs, at least from human UCB, are the more primitive and more quiescent HSC subset, possessing the ability to differentiate into CD133+CD34+ repopulating HSCs, although they may also be lineage biased or primed as they can bypass this latter lineage trajectory and differentiate directly into megakaryocytic–erythroid progenitors (MEPs).

As well as defining human HSCs, major efforts over the past decades were made to determine their pathways and mechanisms of lineage commitment and differentiation into mature functional hematopoietic end cells via increasingly committed HPCs as reviewed by Doulatov et al. [131]. Accordingly, and as described below, a classical hierarchical or pyramidal model of human adult hematopoiesis and revisions of this model were developed principally by comparison with more extensive research on murine HSCs and their lineage trajectories and differentiation programs as described by others (reviewed in [146,154,155,177,178]).

### 3.4. Hierarchical Models of Human Hematopoiesis

#### 3.4.1. Evolution of the Classical Hierarchical Model

**Figure 3:**
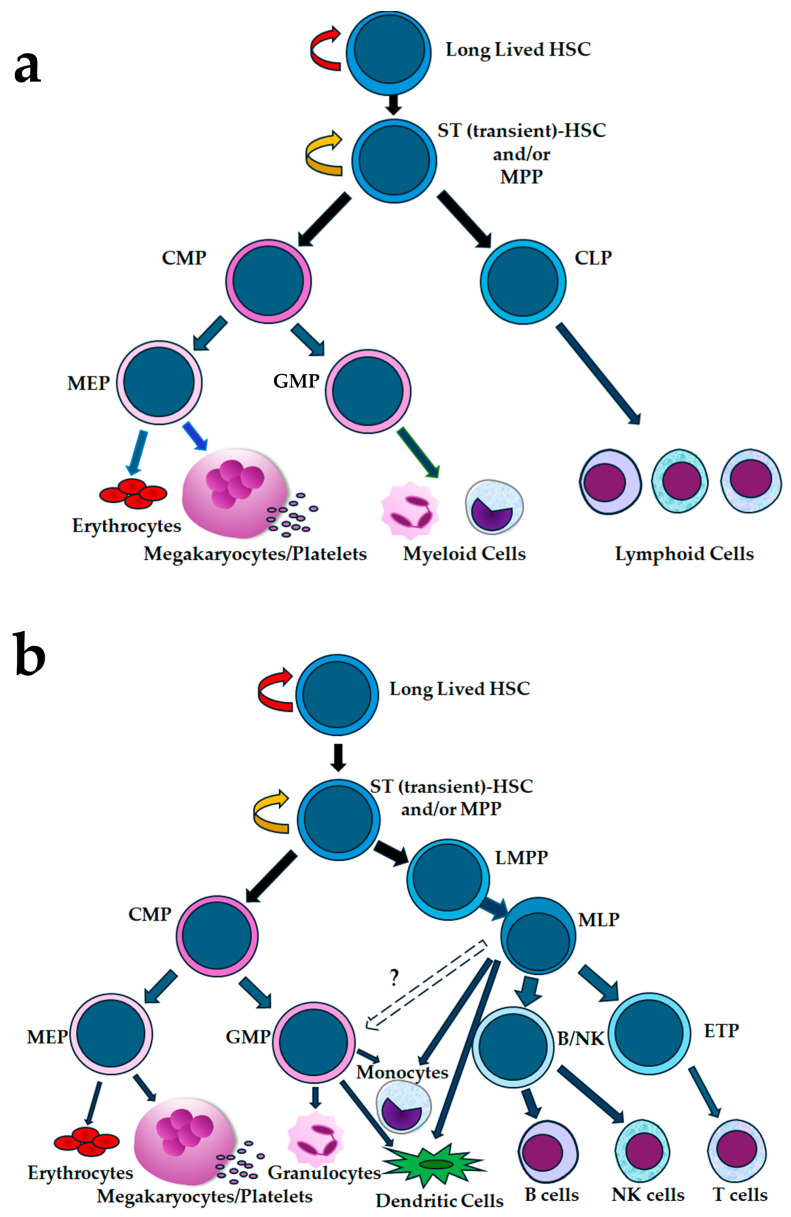
Two Iterations of the Classical Hierarchical Model of Hematopoiesis. (**a**) Early version of the classical model and (**b**) revised classical hierarchical model adapted from Doulatov et al. [131]. In these models, long-lived or durable, self-renewing (red arrow), multipotent CD34+ HSCs give rise to transiently repopulating or short-term (ST) CD34+ HSCs or MPPs with limited abilities to “self-renew” (yellow arrow). (**a**) shows a bifurcation of HSCs into common myeloid (CMPs) versus common lymphoid progenitors (CLPs). CMPs generate megakaryocyte-erythroid progenitors (MEPs) and granulocyte-macrophage progenitors (GMPs), which respectively give rise to erythrocytes and megakaryocytes/platelets or to myeloid cells (including monocyte/macrophages, neutrophils, eosinophils, basophils, mast cells). CLPs generate all lymphoid cells (B, T, NK cells). (**b**) demonstrates that HSC/MPPs segregate into CMPs which generate MEPs, the progenitors for erythrocytes and megakaryocytes/platelets, or GMPs, the progenitors for granulocytes, monocyte/macrophages, and dendritic cells, or into lymphoid-primed multipotent progenitors (LMPPs) with lympho-myeloid potential and then into multilymphoid progenitors (MLPs) that generate lymphoid lineages (T, B and NK cells) but retain myeloid potential (for monocyte/macrophages and dendritic cells). While there is a stronger propensity for MLPs to generate B, T, and NK cells, it is unclear in this model if monocytes and dendritic cells differentiate directly from the MLPs or do so via a GMP intermediate (as indicated by the dashed arrow and question mark). Abbreviations: MPP, multipotent progenitor; CMP, common myeloid progenitor; LMPP, lymphoid-primed multipotent progenitor; MLP, multilymphoid progenitor; MEP, megakaryocytic erythroid progenitor; GMP, granulocyte macrophage progenitor; ETP, early T lymphoid progenitor; B/NK, B lymphoid/natural killer cell progenitor.

The classical hierarchical model of hematopoiesis envisaged deeply dormant HSCs with the greatest self-renewal capacity and longevity, with durable long-term in vivo reconstituting potential and serial transplant ability and with multipotential lineage differentiation potentials positioned at the top of the hematopoietic pyramid and upstream from the shorter-term in vivo reconstituting HSCs, which subsequently branched into phenotypically defined common myeloid progenitors (CMPs) or common lymphoid progenitors (CLPs). These committed precursors gradually gave rise, in a rigid, but stepwise, manner, to even more committed or lineage restricted progenitors, such as megakaryocyte–erythroid progenitors (MEPs), granulocyte–macrophage progenitors (GMPs), and specific lymphoid precursors, before subsequently differentiating into more than 10 specific subsets of mature blood and immune cell lineages (reviewed in [131,154,155,177]) as illustrated in Figure 3a. This differentiation from the HSC is accompanied by a loss of the ability of HPCs to self-renew. Additional studies using human UCB and/or adult BM [66,131,179,180] supported the concept that long-lived HSCs could give rise to ST-HSCs and MPPs, which could segregate into lymphoid-primed multipotent progenitors (LMPPs), multilymphoid progenitors (MLPs) with lymphoid (B, T, and NK cells) and certain myeloid lineage (monocyte and dendritic cell) potential, and CMPs with myeloid potential, leading to revisions of the classical hierarchical model of hematopoiesis such as that exemplified in Figure 3b.

Further research over the past decade has suggested that, like murine hematopoiesis [181], the human HSC, MPP, and more committed HPC compartments are heterogeneous [148,150,154,155,182]. As exemplars, Notta and colleagues separated human fetal liver, UCB, and adult BM CD34+CD38-CD90-CD45RA-CD49f- MPPs into three subsets (MPP F1–F3), identified as being CD71-CD110{cMPL/BAH1}-, CD71+CD110-, and CD71+CD110+ and distinct from each other and from CD90+CD49f+ HSCs [132]. They also used differential expression of CD71 and CD110 together with other biomarkers to define multiple CMP, GMP, and MEP subpopulations. Their results revealed that the human hematopoietic lineage hierarchies change during development, and, although human fetal liver contained significant numbers of intermediate oligopotent progenitor cells as well as multipotent and unipotent progenitors, in adult human BM multipotent and unipotent progenitor cells predominated [132]. Thus, for adult BM, they proposed an alternative model consisting of a two-tiered hierarchy, with the upper tier comprising lineage-primed HSCs and MPPs and the lower tier comprising unipotent progenitor cells, thus questioning the existence of such intermediate progenitors as CMPs and MEPs [132]. Subsequently, Karamitros et al. [183] directly compared the composition and potency of human UCB enriched for LMPPs, MLPs, and GMPs using scRNA-seq analyses, functional in vitro assays, and an in vivo humanized immunodeficient mouse ossicle model to define lineage output and demonstrated that these subsets were distinct from one another both functionally and transcriptionally but marked heterogeneity was evident within each subset at the single cell level. Although they identified rare multilineage progenitors, such as LMMPs with both lymphoid and myeloid potential, MLPs with B, NK, T and residual monocytic cell potential and GMPs which were principally myeloid progenitors with residual NK and B cell potential, the heterogeneity in lineage potential found in these enriched subsets at the clonal level led these authors to conclude that a continuum of lympho-myeloid progenitors (phenotypically classified as LMPP, MLP and CMP subsets) exists downstream from HSCs, and that decisions to progress towards lymphoid–myeloid differentiation pathways could be taken at multiple levels.

At around this time, Velten and colleagues mapped the progression of young adult human BM HSCs to committed progenitors using single-cell technologies (flow cytometry, scRNA-seq, functional assays), concluding that HSCs acquire lineage biases gradually as they differentiate, a process that did not fully support the classical hierarchical model of hematopoiesis [184]. Instead, it was reminiscent of a continuum model proposed earlier for murine BM stem cells [185]. From their studies, Velten and colleagues proposed the existence, within the CD34+CD38- human BM immature HSPC compartment, of a Continuum of LOw-primed UnDifferentiated (CLOUD) HSPCs from which distinct unilineage cells could emerge. While the immediate progeny of CD34+CD38- HSCs, including the MPPs and MLPs, were viewed as transitory states within this continuum rather than as discrete lineage-restricted immature HPCs occurring at specified hematopoietic branch points, evidence was provided for the existence of discrete unilineage-restricted HPC subsets that coincided with an upregulation in cell surface CD38 expression [184]. The pros and cons of this experimental approach have been reviewed by Laurenti and Göttgens [155] and will not be reiterated here.

#### 3.4.2. Further Heterogeneity of Human Hematopoietic Stem Cells and Their Progeny Revealed with Additional Cell Surface Biomarkers and Single-Cell Genomics

The heterogeneity of the human UCB CD34+ HSC compartment has been examined further by various research groups using additional, albeit limited, cell surface biomarkers. For example, Knapp et al. [186] demonstrated that, while around 7% of lentiviral barcoded UCB Lin-CD34+CD38-CD45RA-CD90+CD49f+ HSPCs could engraft long term (7–9 months) in primary NRGW^41^/W^41^ sublethally irradiated recipient mice, only a limited number were capable of engrafting (for 21–30 weeks) in secondary transplant recipients; the latter cells were CD33+ (Siglec-3+) and also expressed higher levels of CD133. Interestingly, multilineage or unilineage reconstitution was evident in primary and secondary transplant recipients [186]. CD201 (EPCR) is another example of a biomarker expressed on the surface of human UCB CD34+CD45RA- long-term repopulating HSCs, when the reconstituting potentials of these cells (not exposed to UM171 in vitro) were assessed in sublethally irradiated NSG mice receiving serial transplants (24- and 18-week readouts for primary vs. secondary transplants, respectively) [187]. These HSCs were also CD133+ and CD49f med/+ [187]. CD201 was subsequently found to be expressed on c. 30% of CD34+CD38-CD90+ and almost 50% of CD34+CD38-CD90+GPI-80+ human week 16 fetal liver HSPCs and c. 7% and <1% of CD34+CD38-CD45RA-CD90+ human UCB and adult BM HSPCs, respectively [188]. The enriched human fetal liver CD34+CD38-CD90+CD201+ and UCB CD34+CD38-CD45RA-CD90+CD201+ and CD34+CD38-CD45RA-CD90+CD49f+CD201- fractions were then tested and demonstrated multilineage engraftment in sublethally irradiated NSG mice at 16 weeks post-transplant [188]. Given that HSC activity, at least in human UCB, exists in both the CD90+ and CD90- Lin-CD34+CD38-CD45RA-CD49f+ subpopulations [156] and that discrimination between CD49f^med/+^ and CD49f- HSPCs can be difficult to standardize for cell-sorting applications [184], Bonnet’s group replaced the CD90 and CD49f biomarkers with CD201 (EPCR) and used a 12Lin-CD34+CD38-CD45RA-CD201+ selection strategy as an alternative to substantially enrich CD34+ HSCs from unexpanded human UCB and adult BM as assessed by their serial transplant ability at 12-week intervals in sublethally irradiated NSG mice [134] (reviewed in [79]). This CD201+ subset comprised c. 0.2–0.8% and c. 0.03% of human UCB and adult BM CD34+ cells, respectively. Notably, both CD133 and CD33 were highly expressed on the surface of these cells, and a high proportion of these CD201+ HSPCs were multipotent, displaying balanced lympho-myeloid differentiation potential. With high repopulating and self-renewal characteristics, approximately one in three of the purified cells were classified as HSCs [134] (reviewed in [79]). These studies also indicated that CD201+ HSCs were positioned immediately upstream of CD201-negative lymphoid-biased CD90+ cells, giving rise first to CD201-CD90- MPPs and then to MLPs/LMPPs, suggesting the occurrence of lymphoid lineage restriction occurs in the HSC/MPP compartment [134] (reviewed in [79,189]). Considerable lineage priming in human CD201+ HSCs and their immediate progeny was also reported [134].

Other biomarkers have been used to identify differential states of dormancy in human HSCs, thus resembling the dormancy states of murine HSCs (reviewed in [155]). Two biomarkers of note, which are significantly upregulated on human quiescent or dormant long-term reconstituting human UCB and adult BM HSCs, allowing some distinction between these deeply quiescent or dormant human HSCs and more activated HSCs, are CD370 (CLEC9A) and the seven-transmembrane and hyaluronic-acid-interacting G-protein coupled receptor family group 5 member C (GPRC5C) [190]. An examination of CLEC9A expression on human UCB CD19-CD34+CD38-CD45RA-CD90+CD49f+ cells revealed the gradual transition from rare multipotent long-term repopulating CLEC9A^hi^CD34^lo^ HSCs (which exit quiescence slowly) towards lymphoid commitment with the generation of lymphoid-primed CLEC9A^lo^CD34^hi^ cells that are distinct from LMPPs, that show more rapid exit from quiescence and that possess restricted lympho-myeloid potential and infrequent durable reconstituting activity [190]. By comparing scRNA-seq profiles, Zhang et al. [190] demonstrated upregulation of the quiescence-associated *CLEC9A* gene and downregulation of the *CDK6* and *MYC* cell cycle genes in adult human BM CD34+CD38-CD45RA-CD90+ CD49f+ long-term repopulating HSCs as opposed to CD34+CD38-CD45RA-CD90-CD49f- short-term repopulating HSC subsets. Expression of the GPRC5C cell surface molecule was also organized along a gradual cell dormancy axis, with its upregulation on human UCB and adult BM HSCs associated with a higher content of G_0_ and long-term multipotent repopulating cells (measured 24 or 36 weeks, respectively, after primary transplantation into sublethally irradiated NBSGW), and delayed cell cycle entry when compared to GPRC5C-negative HSCs [190].

There has generally been a disconnect when relegating phenotypically defined immature HSPCs to discrete subsets using a limited number of cell surface biomarkers and flow cytometry and comparing these with cell states defined by scRNA-seq-based transcriptomic profiling. One approach to reconciling these data has been in the development of antibody-derived tags (ADTs), whereby Mabs to over a hundred cell surface epitopes or isoforms can be individually tagged with barcoded oligonucleotides and their binding to single HSPCs assessed concurrently with single-cell transcriptomic profiling, thereby providing a high-resolution map of single HSPC states.

**Figure 4:**
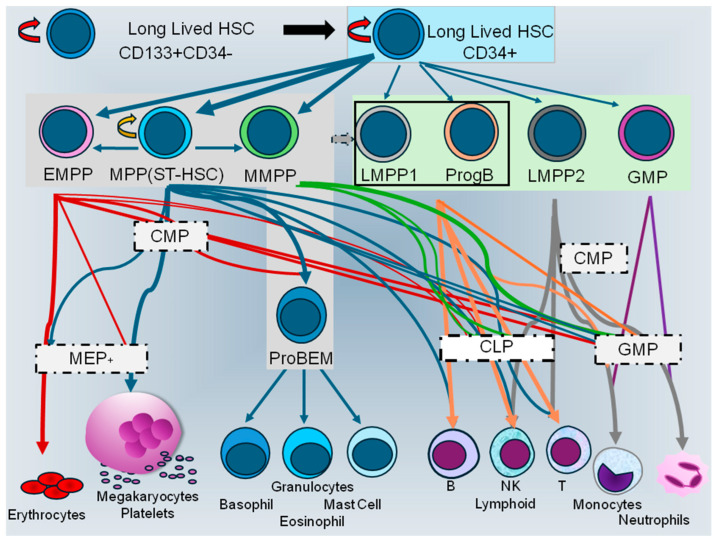
Schematic Representation of the Updated Phenotypic Heterogeneity of Young Adult Human BM CD34+ HSPCs, Particularly in Relation to Multiple MPP and LMPP Subsets and their Lineage Potentials. Adapted from the preprint of Ediriwickrema et al. [191]. The CD34-CD133+ HSCs represent rare cells that are thought to be more immature than and give rise to CD133+CD34+ HSCs, with both HSC subsets depicted as the more extensively self-renewing (indicated by red arrow) long-lived (LT) HSCs. These mostly reside in a quiescent (G_0_) state in young human adult steady state bone marrow but may exhibit considerably more heterogeneity in terms of their depth of quiescence and their self-renewal and lineage potentials than shown here, especially as adults age. When activated, the LT-HSCs may directly generate multipotent progenitors (MPPs) or ST-HSCs with the potential for more limited self-renewal (indicated by yellow arrow) and to differentiate into all lympho-myeloid lineages. LT-HSCs may also directly generate erythroid-biased MPPs (EMPPs) and myeloid-biased MPPs (MMPPs). Alternatively, these more restricted MMPPs and EMPPs may derive directly from the multilineage MPPs themselves. The EMPPs are to some extent reminiscent of platelet–erythroid–myeloid-biased HSCs described in murine bone marrow although their preference is to differentiate into erythrocytes and, to a lesser extent, into platelets, granulocytes, neutrophils, and monocytes. Platelet-biased HSCs, which have multipotent potential, but which directly and preferentially generate platelets when the need arises (via lineage bypass pathways), have been described in murine adult bone marrow, becoming more prominent as mice age. These were not described in the preprint from which this model was adapted [191] and hence are not depicted here. However, platelet-biased HSCs have been described in regenerating human bone marrow and this is discussed in Section 3.8.2. LT-HSCs may also generate lymphoid-primed (L) MPPs, B lymphoid progenitors, and GMPs. Of these, LMPP1 and B lymphoid progenitors (ProgB) possess the strongest lymphoid potential and lesser ability to generate neutrophils and monocytes, while LMMP2 possesses lympho-myeloid (T, NK, neutrophil, monocyte) potential, and GMPs differentiate into neutrophils and monocytes. Blue box: long-lived CD34+ HSCs are phenotypically defined as Lin-CD34+CD38dim/-CD45RA-CD90+. Grey box: 4 distinct subsets within the phenotypically defined Lin-CD34+CD38dim/-CD45RA-CD90-CD2- HSPC compartment, segregated by expression of CD69 and CCL1 into CD38dim/-CD69+ MPP/ST-HSC (MPP1), CD38dimCD69- erythroid-biased MPP/EMPP (MPP2), CD38dim/-CLL1+ myeloid-biased MMP (MMPP), and the CD38dim/-CD69-CD117+ basophil, eosinophil, and mast cell progenitor (ProBEM). Green box: the Lin-CD34+CD38dim/loCD90-CD45RA+ LMPP compartment is further segregated into CD45RA+CLL1- LMPP1, CD45RA++CD19dim B cell progenitors (ProgB), CD45RA++CD2+ LMMP2, and CD45RAdimCLL1+GMP. White boxes with dashed outline represent previously defined CD34+CD38+ CMP, GMP, CLP, and MEP compartments that were not assessed in the studies described [191].

In a recent preprint [191], Majeti’s group has used high-resolution scRNA-seq concurrently with scADT-seq to profile healthy adult human BM HSPCs. From this, they selected additional cell surface biomarkers to enrich immature HPSC subsets and analyzed them functionally by using clonal in vitro and low-dose in vivo primary transplant assays. Notably, human adult BM MPPs, that were detected in the CD34+CD38dim/-CD90-CD45RA-CD2- cell fraction, could be divided into four subsets based on differential CLL1 and CD69 expression patterns. The CLL1-CD69+ ST-HSC or MPP1 subset possessed long-term hematological repopulating potential (measured 17–18 weeks after transplantation into sublethally irradiated NSG mice) and multilineage lympho-myeloid differentiation potential (for seven hematopoietic lineages), while the MPP2 or CLL-CD69- erythroid-biased MPP (EMPP) and CLL1+ myeloid biased MPP (MMPP) showed reduced in vivo hematopoietic repopulating potential, but were distinct from CD69-CD117+ basophil, eosinophil, and mast cell progenitors (ProBEM). In contrast, the CD34+CD38dim/-CD90-CD45RA+ enriched LMMPs, which possess different lympho-myeloid potentials, were segregated into CD2-CLL1- LMPP1 and CD45RA++CD19dim B cell progenitors (ProgB) with high lymphoid potential, CD45RA+++CD2+ LMPP2 with low lymphoid potential, and the CD2-CLL+ GMP subset with neutrophil and monocyte/macrophage potential. These MPP and LMPP enriched subsets were distinct from the more differentiated CD34+CD38+ CMP, CLP, MEP, and GMP subsets described by others. The proposed relationships and diverse lineage outputs or priming of these MPP, LMPP, B lymphoid progenitor, and GMP subpopulations are illustrated in Figure 4. This new classification of human MPPs and LMPPs contrasts with the 4–6 myeloid- or lymphoid-biased MPP subpopulations detected in mice (reviewed in [32]) and which show little concordance with their putative human equivalents [191]. From their studies, Ediriwickrema and colleagues [191] concluded that their new flow cytometric strategy did not purify adult human BM MPP and LMPP subpopulations to homogeneity, but rather enriched for a gradient of cell states present in the immature HSPC compartment.

#### 3.4.3. Human Adult BM Hematopoiesis: A Continuum, a Punctuated Continuum, or a Series of Discrete Meta-Stable States?

As indicated above, advanced single-cell technologies (reviewed in [192,193]) have revealed that the human HSPC compartment is more heterogeneous and that human hematopoietic lineage specification and differentiation trajectories appear much more complex and fluid than otherwise imagined, therefore challenging the classical hierarchical model of hematopoiesis. This has led to two alternative models. The first, the continuum model, proposes that hematopoiesis in healthy young adult human BM is a continuous process, with a continuum or gradient of undifferentiated low-primed immature HSPCs differentiating directly into unilineage HPCs rather than transiting through discrete intermediate multipotent or oligopotent HPCs [184]. The second, the punctuated continuum model, incorporates continuous differentiation within the immature HSPC compartment with hierarchically structured transition points, represented functionally by the presence of discrete cell subsets [132,186,194] (reviewed in [146,155,195]).

Recently, Zhang et al. [194] used the Cellular Indexing of Transcriptomes and Epitopes by Sequencing (CITE-Seq), combining this with Infinity Flow and the scTriangulate machine learning computational platforms, to generate concurrent high-resolution transcriptomic and (with a carefully optimized panel of 132 ADTs) cell surface proteomic profiles. They identified over 80 distinct cell subsets (encompassing the immature CD34^hi+^ HSPCs, mature immune cell subsets, and stromal cell subpopulations) in healthy adult human BM, representing the most detailed map to date [194]. It should be noted that a subset (37 of 132) of the ADTs provided consistent and reliable results across the donor samples tested, with a notable exception being CD45RA and with the caveat that more diverse donor samples require testing. Their data nevertheless identify new HPC subsets, more clearly demarcate and further enrich for other subsets, such as MEPs (based on CD133 expression), and strongly support the concept that adult human BM HSPC differentiation proceeds “*through a series of discrete meta-stable states*” with restricted multipotency and/or unipotency, rather than a continuum of cell states (reviewed in [195]). These evolving models provide HSPCs with the opportunity to switch in a timely manner, via flexible transcriptional programming that is epigenetically regulated, from steady state hematopoietic cell production to the generation of hematopoietic cell subsets that respond appropriately to different stressors. However, most of the studies cited above that use single-cell transcriptomics or assess clonal fate describe a static snapshot of human hematopoiesis. As an alternative to transplantation, more recent studies have espoused the use of barcoding or lineage tracing without transplantation to determine individual HSPC lineage fates that are more closely linked to steady state hematopoiesis.

### 3.5. Can Lineage Tracing Distinguish Homeostatic from Perturbed or Regenerative Adult Hematopoiesis?

#### 3.5.1. Lineage Tracing in Surrogate Models

Lineage barcoding has principally been used to assess the clonal behavior of murine HSPCs in vivo over time, providing information on their potency or lineage contribution, activation status, and longevity. To do so, genetic features are tracked either prospectively (i.e., via those introduced exogenously) or retrospectively (i.e., via those intrinsic to the organism being studied). In the former case, murine HSCs have, for example, been tagged with inducible and heritable knock-in reporter transgenes and viral vectors or by using the RNA barcoding *PolyloxExpress* [196] or the inducible CRISPR array repair lineage tracing (CARLIN, a CRISPR-based scarring method) systems [197]. The latter two integrate clonal cell fate/lineage relationships with the transcriptomic signatures of individual cells and possess the ability to identify both differentiation inactive and active multipotent HSCs (reviewed in [146,196,197]). The functionality of the barcoded cells is then assessed in vivo and in vitro.

Some lineage-tracing experiments have relied on transplanting barcoded murine HSC clones into recipient mice to test functionality, but this can strongly accelerate erythroid and neutrophil/monocytic–dendritic cell differentiation, which is not reflective of the real-time dynamics of the HSPC differentiation under steady state conditions (see, for example, [107,146,177,178,198,199,200,201,202,203,204,205]). On the other hand, barcodes induced in vivo mitigate the effects of transplantation (reviewed in [177]). Interestingly, Lu and colleagues [206] have shown uniform self-renewal and differentiation (without lineage bias) of all engrafted barcoded murine HSCs following non-conditioned transplantation, a process which they propose minimally perturbs hematopoiesis and simulates steady state conditions. This contrasted with transplantation after irradiation-induced myeloablative conditioning where a small number of dominant murine HSC clones differentiate and exhibit lineage bias [206]. CARLIN mice have been used to analyze the perturbation by 5-FU of adult murine hematopoiesis at the clonal level. Results indicate that only a small number of highly active or cycling HSCs regenerate the blood system after 5-FU myeloablation, while there was a minimal contribution of murine HSCs to steady state or unperturbed hematopoiesis at least over a 20-day experimental assessment period [197].

Murine studies, which integrate single-cell transcriptomic results with label propagation studies using barcoded HSPCs and where self-renewal and differentiation rates can be assessed across the continuum of transcriptional data, suggest that the transcriptomic expression data correlate poorly with the dynamics of HSPC differentiation, while the differentiation of HSPCs is enhanced by HCT [204]. In a recent preprint where various enriched barcoded murine HSPCs (long- and short-term HSCs, LMPPs, CMPs, CLPs, and MDPs or macrophage–dendritic progenitors) were compared in transplant studies (up to 115 days), Lin and colleagues [205] have also failed to find a correlation between their transcriptional profiles and functions in vivo, where clonal fate patterns varied widely. They argued that it was difficult to distinguish between the continuum model of Velten et al., the acquisition of continuous and gradual lineage priming, or if biased HSPCs with differing fates or potencies differentiate along multiple tracks, although their studies favored the latter [205]. Because of limited barcode diversity of the Cas9/CARLIN murine line, Li and colleagues [207] have further developed this mouse line into an inducible Cas9-TdT lineage-tracing murine line termed DARLIN, which generates massive barcode diversity. This, together with the Camelia-seq multiomics sequencing tool, concurrently integrates data on cell lineage and epigenomic (chromatin accessibility, DNA methylation) and gene expression profiles for individual cells.

#### 3.5.2. Lineage Tracing and Adult Human Hematopoiesis

It is obvious that the clonal lineage-tracing approaches above are not generally applicable or translatable for analyzing steady state human hematopoiesis. Where they are used, the functionality in vivo of enriched human HSCs is often assessed, after their in vitro culture and genetic barcoding, by their capacity to reconstitute hematopoiesis after transplantation into irradiated/myeloablated or non-conditioned immunodeficient mice. Such an approach suffers from the limitations associated with xenograft models and appears more reminiscent of regenerative or perturbed hematopoiesis than of homeostatic or steady state hematopoiesis (reviewed in [146]). The latter also applies to the longitudinal tracing of proviral integration sites in engrafted hematopoietic cells after autologous CD34+ HCT for patients receiving gene therapies as described earlier (reviewed in [112]). Furthermore, these therapies are relatively rare and, when transplanted in humans, use bulk rather than single HSPCs for transplantation. In recent HCT studies on non-human primates, which have a close evolutionary relationship to humans and which more closely resemble human HCTs than do rodent or human–rodent xenograft models [208,209], Radtke and colleagues [210] have tracked the clonal dynamics of transplanted lentiviral-barcoded CD34+CD45RA-CD90+ enriched HSCs (from and in young pigtail macaques) for up to four and a half years after myeloablative HCT. Their results suggest the contribution of rapidly expanding multipotent HSC clones to early neutrophil recovery post-transplant, with individual HSC clone frequency then declining over time before stabilizing about one year after transplant when fewer symmetrically expanding HSC clones contribute to long-term repopulation [210]. The contribution of other progenitors such as MPPs was not assessed, but these studies challenge the view that persisting long-term repopulating HSCs rarely divide and do not contribute at the clonal level to both the early and late phases of hematopoietic reconstitution. Given these are transplant studies, the results are likely to reflect the plasticity of HSCs during regenerative hematopoiesis or as an emergency response to myeloablative-driven transplantation, with HSPCs bypassing cell division and/or default lineage commitment pathways and rapidly prioritizing the production of specific or unilineage mature hematopoietic cells (reviewed in [178]).

Retrospective single-cell lineage tracing is a promising aid for assessing the dynamics of adult human hematopoiesis and may avoid some of the hematological stressors associated with prospective barcoding such as HSPC ex vivo manipulation (HSPC collection, enrichment, and ex vivo genetic engineering/editing) and patient conditioning and HCT. These endogenous “barcodes” include germline nuclear and mitochondrial DNA variants that occur naturally from conception and somatic DNA mutational signatures and heritable epigenetic alterations which may accumulate throughout life (see recent reviews on genetic variation by [211,212,213,214,215]). The advances in, and pros and cons of, these lineage-tracing strategies have been discussed in detail elsewhere (see, for example, [146,198,211,212,213,214,215]) and, except for some recent exemplars below, will not be reiterated in detail here.

Lineage relationships have been inferred from an analysis of somatic mutations based on whole genome sequencing of HSPC clones grown in vitro. Following on from earlier studies [216,217], Mitchell and colleagues [218] carried out whole genome sequencing on human UCB, PB, and BM Lin-CD34+CD38-CD45RA- enriched HSC/MPP clones (21 days after culture in medium designed to promote erythromyeloid and NK cell growth). Cells were sourced from a small cohort of donors of varying ages (10 individuals with an age range of 0–81 years). The phylogenetic trees derived indicated the highly polyclonal and stable contribution of 20,000 to 200,000 enriched HSCs/MPPs to blood formation in adults under 65 years old, reducing to an estimated 10 to 20 enriched HSCs/MPPs in adults over 75 years, thereby demonstrating decreasing hematopoietic diversity in the elderly [218]. Concurrently, Fabre and colleagues [219], using a targeted genomic sequencing approach, sequentially tracked hematopoietic clones in the PB of a large cohort of older human donors (over 54 years old) over periods ranging from 3.2 to 16 years. Single cultured PB erythromyeloid colonies from these donors were sequenced and somatic mutations used as barcodes to construct phylogenetic trees. They reported an initial stable exponential growth rate for most hematopoietic clones which preceded the development of oligoclonal or clonal hematopoiesis in donors of increased age; the latter was dependent on the driver gene mutations detected [219] (reviewed in [220]). Importantly, attempts are now being made to identify the many unknown driver mutations which enhance such clonal hematopoiesis and to determine at which stage in ontogeny these driver mutations arise (see, for example, [221]).

Given that nuclear DNA variant detection by single-cell whole genome sequencing lacks cell state information (see [107,146]), mitochondrial DNA variants have served as alternative barcodes. Potential limitations or confounders of this approach include mitochondrial mutations that affect HSPC clonal and cellular fitness, horizontal transfer of mitochondria between cells, variable mitochondrial content in cells (with none present in human erythrocytes), and variable mitochondrial DNA copy number (usually 1000 to 10,000 copies per human cell) reflecting bioenergetic requirements; additionally, mitochondrial mutations may exist in all or in a subgroup (homoplasmic or heteroplasmic mutations, respectively) of the individual cell’s mitochondrial DNA copies (reviewed in [211,222]). Of further note, recent studies by Weng et al. [223] combine deep single-cell mitochondrial mutation profiling (ReDeeM) with single-cell multiomics (using scRNA-seq and scATAC-seq to measure transcriptomics and chromatin accessibility, respectively) to define lineage output from, and the physiological state of, individual CD34+CD45RA-CD90+ enriched HSPC clones sourced from a limited number of young adult human BM donors. They reported that most HSC clonal groups actively contributed to steady state hematopoiesis, albeit with moderate HSC variability or bias in lineage output, and that functional HSC heterogeneity was stable in terms of total HSC lineage output and lineage biases when sampled sequentially over a number of months [223]. In contrast to the balanced polyclonal contribution of HSCs to hematopoiesis in young human adults, the phylogenetic trees of two older adults (over 75 years old) displayed a more oligoclonal structure, indicating a decline in hematopoietic diversity with aging [223]. In a recent preprint, Campbell et al. [224] reported that the mitochondrial complement of adult human HSCs is replaced between two- and ten-fold more rapidly than the cells divide or every 4–19 weeks. They argue that heteroplasmic drift in somatic mitochondrial variants and independent mitochondrial mutation convergence may considerably alter the effectiveness of mitochondrial DNA mutations as lineage markers in single-cell sequencing studies. In another recent preprint, Scherer et al. [225] have developed an alternative method for lineage tracing, termed Epi-clone. This is a single-cell droplet-based method that is targetable, scalable, and measures cell-specific and heritable epigenetic marks or epimutations during HSPC differentiation. These epimutations are reported to be stable over many cell divisions and are heterochromatic and late-replicating domain enriched and principally originate from a fully methylated default state [225]. The Epi-clone method was initially tested with murine HSPCs and then further adapted to human hematopoiesis. An age-related hematopoietic oligoclonality or decline in human BM HSPC clonal diversity was observed. Further advances in optimizing human hematopoietic lineage-tracing methodologies are thus keenly awaited.

### 3.6. Are All BM Sites Created Equal?

Under normal homeostatic conditions, adult human hematopoiesis occurs principally in the BM of the axial skeleton, which includes the vertebrae, ribs, sternum, cranium, scapula, clavicle, ilium, and the proximal epiphysis of the long bones, where HSPC fate is regulated by both intrinsic programs and the microenvironmental niches in which they reside or come into contact (reviewed in [32,34,146,226,227]). Whether these anatomical sites contribute equally to human hematopoiesis under steady state or perturbed conditions is not fully understood, nor is it known if human HSPCs distribute uniformly across the skeletal hematopoietic sites post-HCT.

The organization of the BM niche has been studied in detail in murine models and to a lesser extent in humans (reviewed in [178,228]). However, differences in the role in hematopoiesis of different BM sites across the skeleton have received less attention and have principally relied on murine models. In this context, Verovskaya et al. [229] used viral barcoding to examine the anatomical distribution of transplanted single LSKCD48-CD150+ enriched HSC clones from young and aged mice for up to 11 months post-transplantation and reported a highly skewed representation in different bones (viz., spine, sternum, and forelimbs, and the left versus the right femur, tibia, and pelvic bone) for both age groups. They noted that HSCs were rapidly redistributed across skeletal compartments with a single challenge of G-CSF [229]. Uneven chimerism was also observed in different bones over 12–16 weeks after transplantation of BM cells sourced from tibias, femurs, and hips of donors into recipient mice [230]. Recent studies, comparing murine BM from different anatomical sites [231], have also suggested that these are not all equivalent to each other (reviewed in [232]). Wu et al. [231] developed strategies to visualize multipotent hematopoiesis, erythropoiesis, and lymphopoiesis in the BM of mice. Under homeostatic conditions, single murine HSCs and MPPs (except MPP2) were distributed across different anatomical skeletal sites and were particularly enriched near megakaryocytes, while lineage-committed HPCs and immature hematopoietic cells were associated with discrete blood vessels (viz., sinusoids for neutrophil, dendritic, and erythroid production sites and arterioles for B lymphoid production sites) forming distinct non-overlapping microanatomical production sites for each major blood lineage. This spatial organization was conserved across the skeleton, and the hematopoietic production sites persisted throughout life [231]. The key anatomical features were maintained in such chronic and acute perturbations as aging, G-CSF treatment, phlebotomy, and systemic bacterial infection but could be rapidly and specifically remodeled depending on the type of perturbation [231]. Interestingly, variable stress responses were identified in different BM anatomical sites; the long bones and sternum differentially responded to G-CSF, whereas phlebotomy, which reduced lymphopoiesis similarly in the sternum, tibia, vertebrae, skull, and humerus, expanded erythroid production in the sternum, tibia, vertebrae, and humerus, but not the skull [231]. Importantly, structures used to generate blood cells under steady state conditions were the same as those used for demand-adapted or stress-induced hematopoiesis, and the lineage-specific production sites were independently regulated, synchronous, and could return to homeostatic levels with resolution of the acute perturbation [231]. Recently, Koh et al. [233] have demonstrated significant differences between bone marrow compartments in the murine skull and long bones (the femur). They show the continual expansion of the calvarial BM, which, in comparison with the femoral BM, resists microenvironmental niche aging and increases its contribution to hematopoiesis throughout adult life. A strong myeloid bias was observed in the murine femoral, but not calvarial, BM HSPCs upon aging. A similar expansion of HSCs also appears to occur in the human skull, but further studies are pending. Additionally, Niizuma et al. [234] compared the distribution of KSLCD150+CD34- enriched HSCs in nine adult mouse BMs sourced from the femur and tibia, alveolar, mandibular ramus, calvarium, spine, sternum, scapula, humerus, and pelvic compartments. They found the highest frequencies of functional multilineage HSCs in the alveolar BM in the mandible, as confirmed in serial HCT studies.

As is apparent and in contrast to murine BM, the spatial organization of human BM is not as well characterized. In one recent study, Bandyopadhyay et al. [235] analyzed BM from fresh femoral heads of older human adults undergoing hip replacement operations and compared these to iliac-crest-derived BMs from negative lymphoma staging samples. In parallel with scRNA-seq, CODEX (codetection by indexing with a 53 Mab panel) multiplexed imaging was used to characterize hematopoietic and non-hematopoietic BM cells to predict their intercellular communication patterns and to define the spatial distribution of 32 cell types at the single-cell level [235]. Multiple cellular neighborhoods, as well as six MSC subsets, two endothelial cell subsets, and one vascular smooth muscle population, were identified. While the Fibro-MSC subset most closely resembled skeletal stem cells, CD90+ MSCs and Adipo-MSCs were more interactive with hematopoietic cells, with the latter potentially representing a niche for early human BM Lin-SPINX2+CD34+ HSPCs [235]. Whether this is a suppressive or supportive hematopoietic niche is unclear. Adult human HSPCs did not show preferential occupancy of osteoblastic or perivascular niches. Additionally, myeloid development was spatially restricted in the adult human BMs examined, with HSPCs in the adipocytic niche generating early myeloid progenitors, which appear to migrate first to a hyper-oxygenated early myeloid arterio-endosteal niche before proceeding to a perisinusoidal niche to mature and prepare to enter the circulation [235]. Further expansion of these studies is awaited.

### 3.7. Do Extramedullary Sites Contribute to Adult Human Hematopoiesis Under Physiological Steady State Conditions?

In the physiological steady state, adult human HSPCs are rarely found outside the BM, although extramedullary hematopoietic sites may include the spleen, liver, lung, brain, intestine, and PB of healthy individuals [236,237,238,239,240,241]. Such extramedullary sites generally function to increase hematopoietic output under stress conditions, particularly, but not exclusively, when the adult BM hematopoietic capacity is compromised [242,243,244]. Concentrating on the more recent research into human extramedullary hematopoiesis, Mende et al. [241] used single-cell molecular approaches to examine the characteristics of matched PB, spleen, and BM HSPCs obtained from adult human organ donors with consent and of PB from consenting living adult donors. They also investigated the in vivo repopulating ability of enriched adult PB HSC/MPPs using limiting dilution transplants into sublethally irradiated NSG mice, assessing outcomes over 8 to 16 weeks after the primary transplant. Their key findings were that, under steady state conditions, (1) “active hematopoiesis” was minimal in the extramedullary reservoirs examined (spleen and PB), with the extramedullary HSCs/MPPs analyzed demonstrating reduced numbers of S-G2-M cycling cells; (2) transcriptional signatures in these extramedullary HSC/MPP subsets were mostly shared especially in relation to increased lineage priming and short-term repopulating HSC/MPP gene expression patterns, thereby differing significantly from those in the BM; (3) most homeostatic PB (circulating) HSC/MPPs expressed CD71, and these were biased towards erythroid/megakaryocytic lineage differentiation, in contrast to those in the BM or spleen; and (4) only rare “long-term repopulating” HSCs were present in the adult PB (as assessed 8–16 weeks after primary transplant into sublethally irradiated NSG mice) [241]). Subsequently, Conrad et al. [238] extended these studies to assess human extramedullary hematopoiesis and HSPCs in adult human lungs sourced from deceased donors with pre-consent for research and compared these to matched adult BM and PB samples. As with the spleen, the adult human lung did not contain significant numbers of actively cycling and differentiating HSPCs. Rare repopulating adult human lung-derived HSPCs (measured 10 weeks after primary transplant into sublethally irradiated NSG-SGM3 mice) were identified, as were megakaryocytic- and erythroid-biased HSPCs [238]. One interpretation provided by these researchers [238] was that the adult circulating HSPCs emerge from the BM and subsequently seed extramedullary tissues such as the spleen, skin, and/or lung, where they have the potential to readily contribute to stress-induced erythropoiesis and/or thrombopoiesis.

More recently, Quaranta et al. [239] compared the phenotypic, transcriptional, and functional characteristics of steady state circulating and age-matched healthy human adult PB and BM HSPCs using state of the art flow cytometry, CITE-seq, and various in vitro and in vivo assays. They found G1 pre-activated HSPCs primed for differentiation were predominant in the circulation, as opposed to a higher proportion of S-G2-M phase HSPCs as well as G_0_ dormant primitive hematopoietic precursors in BM [239]. In the PB, they reported: (1) a reduced number of phenotypically defined in vivo repopulating HSPCs (as assessed 20 weeks after primary transplants into NSGW41 mice), (2) an enhanced frequency of short-term repopulating cells, MPPs, and MLPs, (3) HSPCs with reduced expression of molecules associated with HSPC BM retention and cell cycle control, such as CXCR4 [245], and (4) HSPCs with increased expression of molecules such as the tetraspanin CD82, a reported regulator of HSPC migration and adhesion [246]. Quaranta et al. [239] also compared human organ-resident HSPC transcriptomic signatures using available scRNA-seq datasets for a number of extramedullary tissues (viz., spleen, thymus, lymph nodes, liver, kidney, and lung) and examined in vivo clonal trafficking of HSPCs to different BM sites by viral vector integration site analyses in HSPCs from patients treated more than 2 years earlier with gene therapy. Their results support the view that multiple circulating adult human PB HSPC subsets are primed to patrol and rapidly seed into different extramedullary reservoirs or niches, where they can act as “first responders” by readily differentiating into appropriate hematopoietic lineages in a demand-adapted hematopoietic response [239,240]. Of further note and expanding on the studies of Mende and others, they suggest that a human BM MLP subset acquires a T-lymphoid-committed thymus-seeding progenitor type 1 (TSP1) gene signature (see [247,248,249]) before migrating to, and circulating in, the PB enroute to seeding the thymus [239]. Their additional research indicates that the most primitive circulating human adult HSPC subsets may also contribute to steady state hematopoiesis by supplying progenitors to, or via clonal HSC/MPP exchange at, different and more distant BM sites [239] (reviewed in [240]).

### 3.8. The Functional Dynamics of Long-Lived Hematopoietic Stem Cells in Young and Aging Adult Bone Marrow

The hematopoietic system under physiological steady state conditions in the young human adult is dynamic and resilient, constantly adapting to daily demands to remove senescent hematopoietic cells and to replenish them, while retaining the ability to flexibly respond to a variety of stressors as these arise. The replenishment of each hematopoietic cell type varies over time, but the goal is to maintain the appropriate proportions and relatively stable numbers of each hematopoietic cell type (see Figure 2). The lifelong replenishment of mature hematopoietic cells during steady state and regenerative hematopoiesis in adult bone marrow is ultimately dependent on the existence of long-lived HSCs with extensive self-renewal potential.

The HSC compartment has been more thoroughly investigated in mice than in humans, with murine HSCs initially being defined by their ability to serially reconstitute multilineage hematopoiesis following their transplantation in vivo (reviewed in [181,250]). Various studies have shown that the reconstitution potential of these HSCs in vivo following HCT correlates with the depth of quiescence, the time to cell cycle entry, the length of the cell cycle, and cell division frequency [154,181,251,252,253,254,255,256,257,258,259,260]. Thus, depending on their reconstitution kinetics after HCT, murine multipotent HSCs were reported to possess long-term (LT), intermediate-term (IT), or short-term (ST) repopulating potentials, with LT-HSCs exhibiting the most robust and durable multilineage repopulating and self-renewal characteristics [154,181,251,252,253,254,255,256,257,258,259,260]. These and further observations led to the arbitrary classification of HSCs as LT-HSCs, ST-HSCs, and MPPs, based on stepwise declines in self-renewal potential and lineage potency [131,155,251,252,253,254,255,256,257,258,259,260].

To better understand their functions, attempts were made to purify murine HSC and MPP subsets. LT-HSCs from healthy young adult mouse bone marrow were substantially enriched by selecting, from the Lineage-Sca-1+c-Kit+ (LSK)-defined cell fraction, cells that are positive for cell surface biomarkers that include CD150, CD38, CD201 (EPCR), and/or EMCN and those that show low to negative expression of other cell surface biomarkers, such as CD48, CD135(Flk2/Flt3), and CD34 [181,251,261,262,263,264,265,266,267,268,269,270,271]. Different combinations of these biomarkers also allowed the segregation of various subsets of murine HSCs and MPPs from each other (reviewed by [181,258,268,271]). Although these enrichment strategies do not purify HSC and MPP subsets to homogeneity, early studies showed that the LSKCD150+CD48-CD34- subset isolated from healthy young adult mouse bone marrow contained virtually all the serially repopulating LT-HSCs (assessed at 12 + 15 weeks in serial transplants in the presence of feeder cells) [251,260,272]. On average, around 70% of this LT-HSC enriched subset was quiescent (in G_0_), with the remaining cells being mostly in the G1 phase of the cell cycle and with <2% actively cycling (in S+G2/M); these cells were also mostly CD135- [251]. The MPPs, in contrast, were found, in this study, to express cell surface CD34 [251]. By combining this enrichment protocol with in vivo label retention assays (using BrDU and histone H2b-GFP) and mathematical modeling studies, Wilson and colleagues [251,252,273,274] identified at least two cell subpopulations in the quiescent LSKCD150+CD48-CD34- defined HSC subset differing in their divisional frequency or history. The first, identified as the H2b-GFP label retaining HSCs and representing about 15–30% of these phenotypically defined quiescent HSCs, was found to reside in a deeply quiescent or dormant state, where they were less responsive to activation and very rarely divided under homeostatic conditions (about every 145–193 days). The remaining 70–85% of the quiescent cell subset was categorized as homeostatic (or “active”) HSCs that divide approximately every 28–36 days [251,252]. Both cell subsets repopulate hematopoiesis by week 15 after transplantation into primary murine recipients, but the dormant HSCs contain the majority of the serial multilineage long-term reconstitution activity (assessed at 15 + 15 weeks post-HCT) and therefore possess the highest self-renewal potential [251,255]. Heterogeneous divisional histories were observed in the latter case [251]. Dormant HSCs in young adult mice can to some extent be distinguished from the quiescent homeostatic HSCs by their expression of additional cell surface molecules, such as CD38 and EMCN [266,267,275]. The heterogeneity of the phenotypically enriched adult human HSC and MPP subsets is also evident as discussed earlier in Section 3.3, Section 3.4 and Section 3.5 and is further discussed below.

#### 3.8.1. Do Dormant Long-Lived Hematopoietic Stem Cells Contribute to Steady State or Perturbed Hematopoiesis?

The contribution of young adult HSCs to replenishing hematopoietic lineages under homeostatic conditions has been a matter of debate. While some research supports an active contribution of murine HSCs to steady state hematopoiesis [276,277,278], other lineage-tracing or fate-mapping studies suggest that, under homeostatic conditions or in response to mild to moderate acute inflammation or other stressors, murine hematopoietic lineages are replenished principally via MPPs and minimally from LT-HSCs [198,279,280,281,282,283]. It has been argued that the former conclusions are confounded not only by the rarity and heterogeneity of the HSC pool but also by the differential labeling of HSC subsets by the reporter genes (e.g., *Tie2*, *Fgd5*, and *Pdzk1ip1*) used in these lineage-tracing/fate-mapping experiments (reviewed in [178,258,275]). Attempts have been made to reconcile these different studies [200,284].

A prevailing view based on murine studies is that, given dormant or deeply quiescent LT-HSCs divide only about 4–5 times over a 2-year murine lifespan or about once every 5–6 months, and possess the highest self-renewal and serial long-term reconstitution potential [285,286,287], they are more likely to represent a reserve long-lived HSC pool that does not generate significant numbers of hematopoietic cells, or does so at very low rates, under steady state conditions [251,252,284,288]. This dormancy promotes the survival, integrity, and potentiality of these LT-HSCs by protecting them from external, albeit moderate levels of, stress and from the risk of somatic mutations [289,290,291,292]. This in turn prevents their proliferative exhaustion and preserves the most primitive LT-HSC pool with the highest self-renewal and long-term repopulating abilities over a lifetime [291]. However, when needed, these dormant LT-HSCs will respond rapidly to conditions of severe stress that may challenge hematopoietic and organismal survival, such as myeloablation or lethal irradiation [178,181]. Once activated, these dormant LT-HSCs are positioned to proceed with symmetrical or asymmetrical cell division and thereby to self-renew and/or to re-enter the quiescent state, thereby preserving their numbers; alternatively, they may choose to differentiate into all or selected hematopoietic lineages [178,251,252].

Recently, using a modified *Fgd5*-driven lineage-tracing protocol, Munz et al. [283] have confirmed that young murine adult LT-HSCs respond to severe stressors (viz., irradiation or 5-FU administration) by increasing their differentiation into HSCs with low transplantation potential. However, they could not recapitulate such an HSC differentiation response in situations of acute inflammation or blood cell loss or by the administration of certain cytokines/reagents that mediate inflammatory responses or regulate hematopoietic cell generation (e.g., using defined limited dosing schedules of agents such as LPS or polyinosinic: polycytidylic acid (pIpC), which induces interferons, or G-CSF, or depletion of erythroid cells or platelets. Using *Tie2*-driven lineage tracing (which may preferentially target dormant LT-HSCs with balanced outputs), Fanti et al. [282] recently reported that primitive HSCs do not show enhanced differentiation into mature blood cells in response to sepsis nor following depletion of erythroid cells, granulocytes, or B lymphocytes. However, they reported that CD135 (Flt3)+ MPPs are mainly responsible for the sepsis-induced hematopoietic regenerative response, which includes MPP amplification and their rapid differentiation into granulocytes and monocytes. Interestingly, in response to sepsis, both LT-HSC proliferation and apoptosis increased [282], processes that may co-operate with each other to preserve LT-HSC numbers and integrity.

It has been estimated that human LT-HSCs, like those in mice, replicate rarely, but on average around 56 times (range 36 to 120 times) over an 85-year lifespan, with approximately half of these divisions occurring during adult life [293,294,295]. This equates to an adult human HSC replication rate of around once every 1 to 4 years. This is lower than that estimated by Catlin and colleagues, who have calculated an average of one HSC division every 40 weeks (range 25–50 weeks) for adults 18 to 100 years of age [296]. Distinct enriched subsets of human LT-HSCs, that respond differently to regenerative stress, have also been described [293]. As a recent example, two human UCB quiescent HSC subsets at opposite ends of the quiescence (G_0_) spectrum (referred to as latent versus primed HSCs rather than dormant and homeostatic or active HSCs) have been defined by their differential expression of the immune checkpoint ligand CD122 [293]. Deeply quiescent (dormant or latent) human LT-HSCs (defined as CD90+CD49f+CD122^lo^) were found to possess a prolonged cell cycle entry time and increased self-renewal and serial long-term repopulating potentials, while displaying delayed or latent long-term reconstitution kinetics following HCT into immunodeficient mice [293]. In contrast, the CD112^hi^-primed quiescent subpopulation responds rapidly to reconstitute hematopoiesis in primary sublethally irradiated NSG-SGM3 recipient mice. These studies identified significant differences in chromatin states between these two quiescent human HSC subsets. Importantly and as highlighted earlier in this review, Zhang et al. [190] analyzed young adult human bone marrow HSCs, demonstrating that the cell surface GPRC5C receptor expression enriches for dormant or deeply quiescent LT-HSCs with high in vivo hematopoietic repopulating ability but without lineage bias. They also described a continuum of dormant to active human HSC states reminiscent of those identified in the mouse and further showed that a hyaluronic acid (HA)–GPRC5C signaling axis controls this dormancy state in both human and murine bone marrow LT-HSCs [190].

Understanding human HSC diversity is important given potential future therapeutic benefits. One recent example is the application of this knowledge to improvements in cell and gene therapies. By using scRNA-seq, functional in vivo analyses, and a reversible in vitro early G_1_ arrest system, Johnson et al. [139] have shown that human UCB and mPB HSCs may lose LT repopulation ability (independently of HSC cell cycle progression) when subjected (over 6 to 24 h) to specific in vitro culture conditions (developed for ex vivo gene therapeutic or HSC expansion studies prior to HCT) by transiently upregulating stress responses. This process could, to a certain extent, be ameliorated by early inhibition of JAK/STAT signaling by ruxolitinib. On the other hand, if the ruxolitinib-free LT-HSC cultures were continued beyond 24 h, differentiation programs ensued as these LT-HSCs progressed past the early G1 stage of the cell cycle. The development of such approaches may be of therapeutic benefit in minimizing the loss of self-renewal and repopulating functions of HSCs often observed in earlier attempts to manipulate enriched human LT-HSCs ex vivo for gene correction or HSC expansion protocols for HCTs. Other potential benefits of research into the functional dynamics of long-lived HSCs include improving HSC fitness in elderly individuals [297] and will be discussed below.

In summary, current evidence suggests that the principal functions of deeply quiescent or dormant LT-HSCs in healthy young adult mice are to preserve the HSC pool by maintaining their numbers and integrity and to act as a reservoir for lifelong blood/hematopoietic cell production when needed, processes critical to organismal survival. As such, these LT-HSCs may not contribute substantially to the daily steady state production of mature hematopoietic lineages but may have a greater capacity to respond rapidly to conditions of severe stress while maintaining their ability to self-renew and return to a quiescent or dormant state. These LT-HSCs thus appear to persist throughout an adult lifespan, while maintaining their capacity to balance quiescence or dormancy with self-renewal or commitment to differentiate into all defined hematopoietic lineages. Given that dormant and active HSCs have also been described in adult human BM and UCB, it seems likely that dormant or latent human LT-HSCs possess similar functions to those described in mice, but further studies would confirm this conclusion.

#### 3.8.2. Hematopoietic Stem Cell Multipotentiality or Lineage Bias

At the single-cell level and/or based on HCT and lineage-tracing or barcoding studies, current evidence indicates that, while young adult murine and human bone-marrow-derived LT-HSCs principally exhibit balanced multilineage (lymphoid and myeloid) potential, a proportion of HSCs also display heterogeneity in terms of their fate restriction, priming, or bias [178,260,269,275,298]. In particular, research over the past decade or more has described platelet–erythroid–myeloid-biased and platelet-biased durable LT-HSC subsets in young adult murine bone marrow under steady state and transplantation conditions (see, for example, [154,202,257,259,260,268,269,270,275,280,298,299,300,301,302,303,304,305,306,307,308]). By analyzing over 1000 single Vwf+LSKCD150+CD48-CD34- murine bone marrow LT-HSCs in transplant experiments, Carrelha and colleagues [202,303] identified LT-HSCs that preferentially generated platelets and, while generally not giving rise to T and B lymphoid cells, produced smaller numbers of other myeloid and erythroid cells. They also identified platelet-restricted LT-HSCs that, while generating platelets in primary transplant recipients, could also produce low outputs of erythroid cells, granulocytes, and monocytes after secondary transplantation. In contrast, most single Vwf-LSKCD150+CD48-CD34- murine bone marrow LT-HSCs were found to be multilineage, with a balanced hematopoietic (all lymphoid and myeloid cells) or a lymphoid-biased output but lacking platelet bias [303]. Other recent studies show that LSKCD150+CD34-CD135(Flt3)- bone marrow HSCs from healthy young adult mice can be separated into HSCs with balanced myeloid–lymphoid potential or with myeloid bias, based on their differential positivity for cell surface molecule CD150 [309]; the myeloid-biased HSCs make up approximately a third of the CD150+ cell subset and are more highly positive for CD150 (termed CD150^hi+^ HSCs), while the balanced HSCs (termed CD150^lo^+ HSC) have lower CD150 positivity and account for the other two thirds of the LSKCD150+CD34-CD135(Flt3)- enriched HSC population [251,309,310].

A platelet-biased LT-HSC subset (within the Lin-CD34+CD38-CD45RA-CD90+) has also been detected in young adult human bone marrow albeit when assessed in the xenotransplant setting [311,312]. This represents one of six HSC clonal groups within this phenotypically defined subset identified by Aksoz et al. [311] and which displays distinct hematopoietic outputs. Of the other five groups, the main ones include platelet–erythroid-biased, platelet–erythroid–myeloid-biased, and multilineage HSCs. Aksoz et al. [311] showed that stemness and quiescence gene signatures are enriched in the platelet-biased HSCs when compared with multilineage HSCs which demonstrate proliferative gene signatures. They also identified a proportion of Lin-CD34+CD38-CD45RA-CD90+ enriched HSC clones that did not contribute to hematopoietic regeneration after xenografting. Determining if such human platelet-biased LT-HSCs are present and play a role during unperturbed hematopoiesis would be an important step forward in studies such as these, but this would likely be very difficult to assess. In this context, single cell multiomics studies by Weng and colleagues [223,313], that are briefly described in Section 3.5.2, support the concept of HSC diversity in terms of balanced and biased lineage outputs from HSC clonal groups in young adult human bone marrow during steady state hematopoiesis. The existence of lymphoid-biased HSCs has on the other hand been a matter of contention [303,314]. Although further studies are required to resolve conflicting conclusions, current evidence from murine models based on the extensive single-cell transplant (>1000 HCT) analyses described above suggests that lymphoid-biased HSCs are not present in the most durable LT-HSC compartment [275,303]. Some other studies have suggested that a proportion of or most lymphoid-biased HSCs may be more reminiscent of IT-HSCs, rather than either LT-HSCs, ST-HSCs, or MPPs, but further clarity is required [256,259,264,269,297,315,316,317].

#### 3.8.3. HSCs and the Effects of Aging

A great deal of effort has been and is being directed towards defining intrinsic and extrinsic factors that influence HSC function and fitness in young adulthood and how these might change in the elderly [260,318]. Extrinsic factors include the local and systemic microenvironments to which the HSCs are exposed, while specific HSC responses to these microenvironmental stimuli are governed by intrinsic differences amongst HSC clonal groups. A functional decline in HSC fitness is associated with aging, in part due to lifelong stressors (e.g., replicative stress, infections, and inflammatory conditions). This coincides with a numerical increase in the phenotypically defined BM HSC pool but with reduced regenerative ability and self-renewal potential [310,318,319], skewing towards platelet/myeloid-lineage-biased HSCs to the detriment of lymphoid and erythroid lineages [230,311,320], an increase in local and circulating pro-inflammatory mediators (inflammaging), a decline in immunity, a perturbed quiescent state reflecting an enhanced stress response, and a propensity for clonal hematopoiesis [260,297,318,320,321,322,323]. These effects are believed to be driven extrinsically by aging of the bone marrow niche and the presence of a pro-inflammatory environment and intrinsically by the individual HSC genetic make-up, the degree of DNA damage, epigenetic and metabolic changes, diminished autophagy (a process for the removal of damaged organelles and proteins), impaired proteostasis, and alterations in cell polarity [311,318,319,321,322,323,324].

##### Lineage Bias and Aging

Using single-cell technologies, Nogalska et al. [325] recently compared mice of the same chronological age and from the same inbred strain, but with early versus delayed immune aging phenotypes. They found a shift towards myelopoiesis in the former while, in the latter, the observed shift towards lymphoid lineage output was associated with decreased myelopoiesis rather than increased lymphopoiesis. The differences between these two groups of mice were thought to predominantly reflect differences in myeloid output from a small but distinct HSC subset. Increases in transcriptional platelet-priming levels and in the prevalence of platelet-biased HSCs upon aging have been observed in both mice and humans [311,326]. In recent studies using the murine FlkSwitch model, Poscablo and colleagues [327] have identified and lineage traced two HSC trajectories for replenishing murine platelets. In young mice, the canonical differentiation pathway, which originates from Flk2(CD135)- LT-HSCs and proceeds via Flk2(CD135)+ MPPs and their progeny, is multilineage, being capable of replenishing platelets, other myeloid lineages, and lymphoid lineages, while, in aging mice, an alternative (direct, bypass, or shortcut) differentiation pathway originates from Flk2(CD135)- LT-HSCs but has the potential to rapidly generate a distinct subset of megakaryocyte progenitors and platelets without proceeding through other HPC intermediates. This bypass pathway is associated with platelet hyper-reactivity and thrombocytosis [327]. Other recent studies from Carrelha and colleagues (building on their earlier research as described in Section 3.8.2) [202,270,326] have also detected alternative pathways for platelet production from LT-HSCs in young adult mice, one a steady state and slower canonical pathway that progresses through Flt3 (CD135)+ intermediates and another, a rapid emergency activated pathway, that does not. Their results indicate that these two subsets derive from distinct (non-hierarchical) LT-HSC subsets, with the platelet-biased HSCs further expanding upon aging [326]. Aksoz et al. [311] found that the frequency of platelet-biased HSCs in aged human adult bone marrow was significantly increased, while the abundance of multilineage HSCs was reduced. Additionally, it has been suggested that platelets derived from platelet-biased HSCs rather than those replenished via the canonical multilineage pathway may be responsible for vascular thromboses and thrombotic diseases (e.g., ischemic stroke) that increase with aging [327]. If this is so, then the preferential in vivo removal of the former may be of therapeutic benefit [327,328].

Other studies have also reported an increase in murine and human myeloid-biased HSCs relative to balanced multilineage LT-HSCs during aging, thereby reducing lymphopoiesis and concomitant adaptive immune responses and increasing pro-inflammatory myeloid cells [178,297,309,311,329,330,331,332,333]. It has therefore been proposed [309] that the increase in myeloid-biased HSCs during aging is a “*double-edged sword in the battle with novel pathogens, resulting not only in a poor adaptive immune response, but also in detrimental inflammatory responses*”. By selectively targeting cell surface antigens on, and depleting, myeloid-biased HSCs in aged mice, while also targeting KIT and CD47 to block anti-phagocytic activity, Ross et al. [309] sought to rebalance hematopoiesis and to rejuvenate the immune system by preferentially retaining balanced multilineage LT-HSCs. Their results demonstrate that this approach could indeed enhance immunity following vaccination to retroviral infection and viral challenge and reduce pro-inflammatory myeloid cells and hence pro-inflammatory mediators in aged mice. Whether it will be feasible to translate such a therapy to the clinic, however, requires further careful research. In this respect, it is of interest that the three biomarkers, CD150, CD62p, and NEO1, which were used as targets for murine myeloid-biased HSC depletion in the above studies are expressed on a subset of human bone marrow HSCs (defined as Lin-CD34+CD38-CD90+CD45RA-), and efforts are being made to determine if these recognize a human myeloid-biased HSC subset similar to that identified in the murine immune rejuvenation studies.

##### Trained Immunity, Inflammatory Memory, and Aging

A decline in or dysregulated immunity is another feature that accompanies aging and is reflected in increased susceptibility to infections and to chronic viral reactivation, poorer responses to vaccination, and an increased risk of malignancies [318]. The immune system in the adult human or mouse is divided into two main parts, the innate and adaptive immune systems, with both affected by aging. The adaptive immune response, which comprises T and B lymphocytes, is exquisitely specific for, but responds relatively slowly on its first encounter with, a particular pathogen [178,318]. Innate immune cells, on the other hand, include neutrophils, monocytes, dendritic cells, basophils, eosinophils, mast cells, and innate lymphoid cells, which can respond rapidly, but non-specifically, to pathogens and acute inflammatory challenges [178,334], thus representing the first line of defense to pathogens. Cells of the innate immune system can, for example, be recruited to sites of infection, tissue damage, or inflammation, where they work together to mount a rapid response to pathogen infection, promote angiogenesis and tissue repair, and/or inform the adaptive immune system, via antigen presentation, of the infection.

Until recently, it was thought that immunological memory was the domain of the adaptive immune system, whereby T and B lymphocytes, which respond to a specific pathogen, can mount a more rapid and enhanced response to reinfection by the same pathogen. However, this concept was challenged by the discovery of innate immune memory, whereby innate immune cells exposed to a particular pathogen or challenge alter their secondary response to the same or a different challenge [335,336]. This may augment or repress secondary immune responses and constitutes processes referred to respectively as trained immunity or immune tolerance [337,338]. Importantly, while innate immune memory is mediated by mature myeloid cells (termed peripheral trained immunity), various studies suggest that it is sustained by epigenetic reprogramming of HSPCs in response to their exposure to various inducers including inflammatory cytokines, growth factors, LPS, β-glucans, oxidized low-density lipoprotein, and the Bacillus Calmette–Guérin (BCG) vaccine, a process referred to as central immune memory [339,340,341,342,343,344,345,346,347,348,349]. As such, central innate memory is reminiscent of and overlaps the concept of emergency myelopoiesis as elegantly described by Swann et al. [178]. Depending on the nature, strength, and duration of the perturbation, emergency myelopoiesis has also been shown to affect the function of various HSPCs before they return to steady state hematopoiesis, with the most common proposed pathways from murine studies potentially involving i) the activation and proliferation or differentiation of HSCs to aid hematopoietic replenishment, ii) the amplification of myeloid-biased MPP2 and of secretory MPP3 as a myeloid bypass mechanism, iii) the reprogramming of lymphoid-biased MPP4 to dendritic cells, iv) erythroid differentiation inhibition, and v) the formation of GMP clusters via GMP proliferation in the BM microenvironment to further drive myeloid output (as detailed in [178]).

A number of examples of central trained immunity in mice (but by no means all) are given below. As the first example, Kaufmann et al. [347] showed that, in mice, exposure of BCG (a live attenuated form of *Mycobacterium bovis*) to bone marrow (i.e., via intravenous rather than subcutaneous administration) expands murine HSPCs, promotes myelopoiesis, and, in an interferon-γ-dependent manner, educates HPSCs epigenetically to produce trained monocyte/macrophages with increased protection against *Mycobacterium tuberculosis* infection. This is reminiscent of the earlier observation that BCG vaccination can protect very young children against *Mycobacterium tuberculosis* infection, while also decreasing the morbidity and mortality of certain other infections [349,350]. Secondly, De Laval and colleagues [341] showed stable cryptic (phenotypically invisible) epigenetic alterations in murine LSKCD135(Flt3)-CD150+CD48- enriched HSCs following a single LPS challenge and found that this depends on the C/EBPβ transcription factor. This epigenetic priming of myeloid-biased HSCs is then maintained under steady state conditions and may be reactivated rapidly in response to secondary bacterial challenge (e.g., with *Pseudomonas aeruginosa*), when coupled with such metabolic changes as increased oxidative phosphorylation and fatty acid oxidation that marks HSPC differentiation. Thirdly, Mitroulis and colleagues [340,348] reported that β-glucan increased putative myeloid-biased CD41+LT-HSCs and MPP frequency in murine BM but did not affect the overall numbers of enriched LSKCD150+CD48- LT-HSCs 24 h after administration, although the latter LT-HSC enriched subset increased transiently at day 7 post β-glucan administration and returned to normal by day 28. The enriched BM LT-HSCs from day 28 β-glucan-treated mice were transplanted into lethally irradiated congenic mice and enhanced in vivo myelopoiesis was observed 12 weeks post-transplant. Furthermore, β-glucan-induced trained immunity not only enhanced glycolysis and cholesterol biosynthesis in HSPCs but was also able to confer a protective hematopoietic response to a secondary inflammatory or myeloablative stimulus. As another more recent example, Kain et al. [342] found that multiple murine BM HSPC subsets (including HSC and MPP subpopulations) respond transcriptionally to *Mycobacterium avium* infection, and this is accompanied by expansion of a rare, activated HSC subset. Interestingly, these researchers also described durable in vivo protection against secondary *Mycobacterium avium* infection. They then isolated BM c-Kit+ HSPCs from mice one month after initiating *Mycobacterium avium* infection, transplanted these trained HSPCs into naïve sublethally irradiated recipient mice, challenged the recipient mice 6–12 months later with *Mycobacterium avium*, and found enhanced protection of the recipients to this secondary *Mycobacterium avium* infection. In a similar experiment, they also showed some subtle cross-protection via myeloid-biased MPP and GMP against acute influenza A challenge 12 weeks after recipient mice were transplanted with *Mycobacterium avium*-trained adult BM derived c-Kit+ HSPCs. Notably, transplantation of murine Lin-Kit+CD150+CD48-CD34-EPCR/CD201+ enriched LT-HSCs (250 cells per transplant, sourced from murine adult bone marrow one month after *Mycobacterium avium* infection) into naïve sublethally irradiated mice was not sufficient by itself to confer innate trained immunity when such mice were challenged 12 weeks post-transplant with the same pathogen. Collectively, these studies suggest that central innate trained immunity occurs in a subset of HSCs and involves myeloid-biased MPPs and GMPs, but not the most primitive dormant LT-HSCs. Further examples of central trained innate immunity can be found elsewhere [336]. As GM-CSF is a known mediator of emergency myelopoiesis and promotes the development of myeloid-biased HPCs from HSCs as well as the proliferation of GMPs and their differentiation into granulocytes and monocytes in response to infection and inflammation [351], its role in innate immunity was recently examined by Guerrero and colleagues [337]. They showed two effects of GM-CSF, the first one programming GMPs to produce trained macrophages with an enhanced cytokine response and the other programming murine immature LSK+ HSPCs to produce “tolerized” macrophages with a reduced cytokine response, thus potentially limiting the central innate trained memory response and protecting against a cytokine storm. Assessment of the effects of GM-CSF on human bone marrow CD34+CD38- HSPCs versus more mature CD34+CD38+ progenitors (which would include GMPs) suggests that GM-CSF tolerizes the former but promotes trained immunity in the latter [337]. As the human CD34+CD38- HSPCs encompass different HSC, MPP, and HPC subsets, a deeper understanding of the HSPC subsets involved in and mechanisms regulating emergency myelopoiesis and innate trained immunity, especially in elderly humans, is warranted.

A systems biology approach [344,352] has shown impairment of the innate immune response in older human adults, and these and other studies link chronic low-grade inflammation in the elderly (or inflammaging) to such immune impairments [178]. While trained innate immunity is designed to mount a rapid response to a secondary infection, for some infections and chronic inflammatory disorders (e.g., type 2 diabetes, obesity, arthritis, cardiovascular disease, autoimmune disease), where inflammation does not resolve, activation of central trained immunity or emergency myelopoiesis may persist, promoting progression of the disease. A recent example is the development of COVID-19 which may progress to acute respiratory disease syndrome [336,353]. This is accompanied by features reminiscent of emergency myelopoiesis (e.g., myeloid bias, HSC apoptosis, GMP expansion, neutropenia, lymphopenia, anemia) with recent clinical studies demonstrating sustained and pathological activation of inflammatory molecules and dysregulated immune signaling in older adults (as opposed to younger patients) hospitalized with severe COVID-19 (reviewed in [178]). Maladaptive emergency myelopoiesis may also enhance the growth and metastatic spread of some cancers [178].

Recently, Zeng GX et al. [354] have identified, in human UCB, a new HSC subpopulation, which retains memory of previous inflammatory stress and has been termed inflammatory memory HSCs (HSC-iM). These HSC-iMs exist as a subset of phenotypically defined Lin-CD34+CD38-CD45RA-CD90+CD49f+ HSCs, exhibiting enhanced, but distinct, epigenetic and transcriptional signatures related to both inflammatory response programs and LT-HSC dormancy. Their HSC-iM program is enriched in human memory T cells and in older individuals who exhibit clonal hematopoiesis and/or following severe COVID-19 infection, thereby linking inflammatory history with infection, clonal hematopoiesis, and aging [354,355]. Jakobsen et al. [355] further showed that the inflammatory response program is attenuated in human *DNMT3A* and *TET2* mutant HSCs (i.e., those exhibiting loss of function of these epigenetic modifiers) from individuals with clonal hematopoiesis, allowing these more resilient HSCs to resist the harmful effects of inflammation and aging that are detrimental to normal HSCs [324,356,357,358]. However, these mutations can enhance the risk of developing myeloid malignancies [359,360] and can contribute to other age-related diseases [361,362,363] (for comprehensive discussions on clonal hematopoiesis, we refer readers to [364]). It is hoped that understanding these mechanisms in more detail, particularly in humans and reconciling demand-adapted and emergency myelopoiesis with central trained immunity and inflammatory memory, will lead to improved therapies for a variety of diseases that increase with aging as outlined recently [336].

##### Epigenetic and Metabolic Regulation and Quality Control Mechanisms in Hematopoietic Stem Cells and Alterations Associated with Aging

It is well recognized that epigenetic and metabolic statuses play pivotal roles in regulating fate decisions of individual HSCs [275,318,365]. Indeed, epigenetic/chromatin regulators have been described as “the gatekeepers of hematopoiesis” [365]. They have the ability to modify chromatin structure, function, and dynamics in response to changes in the cellular environment, through processes such as DNA methylation, histone post-translational modifications (e.g., by acetylation, methylation, phosphorylation etc.), and remodeling of nucleosomes, with these being influenced by histone variants, non-coding RNAs (e.g., microRNAs, long non-coding RNAs), and 3D chromatin conformation. Without altering the DNA sequence, these epigenetic or chromatin modifiers can control access of regulatory elements to specific regions of the DNA and hence regulate gene expression patterns and eventually cellular functions (reviewed in depth recently in [275,318,365]). The activity of chromatin-modifying enzymes is dependent on essential metabolic cofactors. For example, acetyl-CoA is a donor of acetyl groups for histone acetylation [366]. Further examples have been recently described in more detail [275,318].

There is evidence that epigenetic priming in murine multipotent HSCs occurs prior to expression of transcriptomic signatures and can be selectively maintained or repressed throughout lineage differentiation [298,367,368], while Meng et al. [298] show that differential chromatin accessibility regulates the fate of multipotent and lineage-biased HSCs. Chromatin accessibility profiles are altered in aged HSCs [297,298,369] in response to ongoing or long-term inflammatory stress (inflammaging) [369,370]. Aged HSCs also exhibit changes in DNA methylation and histone modifications related to their self-renewal/proliferative potentials when compared to young adult HSCs [371,372,373]. Interestingly, HSC defects (e.g., phenotypically defined HSC accumulation, myeloid-biased output, and decreased reconstitution capacity) could be reversed epigenetically by deletion of the epigenetic regulator *Phf6* gene in aged mice [374,375,376,377,378]. Thus, delaying epigenetic alterations as individuals age may prove of therapeutic benefit. Changes in epigenetic regulation represent one of the twelve hallmarks of aging defined by López-Otín et al. [379], the others being “*genomic instability, telomere attrition, loss of proteostasis, disabled macroautophagy, deregulated nutrient-sensing, mitochondrial dysfunction, cellular senescence, stem cell exhaustion, altered intercellular communication, chronic inflammation, and dysbiosis*”. A selection of these will be highlighted below as an illustration of other potential pathways that could modify an aging hematopoietic system.

The transition between quiescence and proliferation or differentiation is accompanied by alterations in metabolism, yet whether this occurs uniformly in individual HSCs which display different depths of quiescence or different lineage biases is not yet fully defined, particularly with respect to human adult HSCs (reviewed in [178,258,380,381,382,383,384]). Quiescent HSCs are generally considered to have low metabolic, protein synthesis, and mitochondrial activities despite a high mitochondrial content and to rely principally on anaerobic glycolysis to produce energy, while limiting oxidative phosphorylation and hence reactive oxygen species levels [381,382,384,385,386,387,388,389,390]. It has been proposed that the deeply quiescent or dormant (G_0_) LT-HSCs have the lowest mitochondrial membrane potential, distinguishing these LT-HSCs from active or primed (G1) HSCs which exhibit higher mitochondrial membrane potential [384,389,390,391]. Upon activation, HSCs may enter the cell cycle and complete division in order to self-renew or return to a quiescent state, or alternatively they may differentiate into various hematopoietic lineages [380,392,393,394,395,396]. There is evidence that HSC activation and differentiation are accompanied by increased glucose consumption, glycolysis, mitochondrial biosynthesis, and mitochondrial oxidative phosphorylation and that this establishes a link between glycolysis and mitochondrial metabolism that is not observed in dormant HSCs [381,382,384,389,391,397,398]. Quiescent HSC function and mitochondrial maintenance have also been linked to fatty acid oxidation and lipid metabolism [382,384]. For example, higher levels of mitochondrial NADPH, which are used mainly for cholesterol biosynthesis in HSCs, have been found in murine LT-HSCs with high in vivo regenerative abilities when assessed at the single-cell level than in ST-HSCs [399]. Other recent studies tracing long-chain fatty acid oxidation with [1–^14^C]-palmitate have suggested that LSKCD150+CD48- HSCs from the bone marrow of young and old adult mice possess similar abilities to oxidize these fatty acids, although this is dispensable in young murine adult HSCs but not in normal HSCs from older mice [400]. Merchant et al. [400] proposed that this may be due to a decline in metabolic plasticity with age resulting in the compensatory ability of young, but not old, murine adult bone marrow HSCs to generate acetyl-CoA from pyruvate when long-chain fatty acid oxidation is compromised.

To better define the metabolomes in a heterogeneous subset of enriched HSCs, Cao et al. [401] have recently developed the platform hi-scMet to map metabolomics in individual HSCs. They initially tested this on defined murine HSPC subsets within the hematopoietic hierarchy and demonstrated significant lineage-commitment-related metabolomic changes. Then, using the H2b-GFP label retention approach described earlier, they separated young adult murine bone marrow HSCs into four subsets, termed HSCa, HSCb, HSCc, and HSCd, based on differential and increasing divisional histories and analyzed these using the hi-scMet platform. They described the shift from dormant to active states for the majority of HSCs as representing “a continuous stream-like progression of gradually increased metabolic levels”, which are unaffected by cell size but accompanied by increasing activation of the oxidative pentose phosphate pathway. Significant increases in metabolites, which include glucose, asparagine, ascorbate, arachidonic acid, and 6-phosphoguconic acid (6PG), and a significant decrease in palmitic acid were noted in active (HSCd), as opposed to dormant (HSCa), HSCs at the single-cell level. Concentrating on 6PG (a pentose phosphate pathway metabolite) and on the pentose phosphate pathway, their results show that exogenous addition of 6PG to enriched HSCs ex vivo inhibits differentiation and enhances HPC (myeloid and B lymphoid) reconstitution following myeloablative HCT, while interference with 6PG synthesis in HSCs increases the levels of reactive oxygen species. Further studies on adult human HSCs in young versus old donors using these and other single cell metabolomic techniques, especially when combined with epigenomics and transcriptomics (see details in [382]), would be beneficial and will undoubtedly expand our knowledge in terms of the function and regulation of rare and heterogeneous HSC subsets.

During HSC quiescence, activation, and cell division, mitochondrial quality control mechanisms come into effect to preserve LT-HSC self-renewal ability and other functions. These include the mitigation of mitochondrial oxidative stress [402], the mitochondrial unfolded protein response [403,404], and removal of damaged mitochondria by autophagy (the process whereby mitochondria are engulfed by autophagosomes and then degraded following fusion with lysosomes; also known as mitophagy) [405,406,407,408,409]. Loss of mitophagy and accumulation of aberrant mitochondria by conditional deletion of the *Atg7* gene have, for example, been shown to negatively influence the numbers of young adult murine bone marrow LT-HSCs and their ability to regenerate long-term hematopoiesis in lethally irradiated mice [405]. Mitography is important for preserving HSC self-renewal and quiescence in both murine and human LT-HSCs [407,410]. Quiescence, self-renewal, and erythromyeloid specification in human UCB LT-HSCs, for example, depend in part on transcription factor EB (TFEB) and MYC [410]. Under homeostatic conditions, TFEB is highly expressed in human quiescent UCB LT-HSCs, where it controls the lysosomal degradation of cell surface environmental sensing receptors involved in the activation of LT-HSCs, thereby maintaining quiescence and preserving self-renewal potential. In contrast, MYC represses TFEB-regulated lysosomal programs and drives HSC activation. Various other studies have reported that, in both murine and human LT-HSCs, the asymmetric partitioning or inheritance of mitochondria, autophagosomes, and lysosomes (which fuse with autophagosomes and degrade autophagic contents) can, for example, determine or coincide with the commitment of one daughter cell to differentiate and for the other to self-renew [389,410,411,412,413] (reviewed by [258,381]). Liang et al. [389] found that quiescent murine HSCs with low mitochondrial potential contain or inherit more abundant enlarged lysosomes than HSCs with high mitochondrial membrane potential, while those with fewer lysosomes are thought to be inclined towards differentiation [411]. Increased competitive reconstitution capacity of quiescent HSCs has been associated with the enlargement of their lysosomes following suppression of acidification and associated enhanced mitophagy [389]. In contrast, in relation to HSC symmetrical division [414,415], both daughter cells can inherit defective mitochondria and other organelles [407]. Hence, during aging, HSCs may develop defective mitography, acquire dysfunctional mitochondria, and accumulate reactive-oxygen-species-mediated DNA damage, all of which are detrimental to their function [291,389,405,406,407]. Efforts are therefore being made to avert this HSC decline by enhancing mitochondrial fitness in aged HSCs by drug discovery approaches such as the approach described in [416]. Interestingly, Dellorusso et al. [417] have recently described two distinct states of autography (activated and inactivated) in aged murine HSCs. They demonstrated that suppression of glucose uptake and glycolysis induced by chronic inflammation could subsequently activate autophagy in some aged HSCs, thereby promoting their survival and quiescence and preserving their residual regenerative capacity. Dong et al. [418] identified a role for chaperone-mediated autophagy, a selective lysosomal protein degradation mechanism, as an HSC quality control mechanism that maintains HSC functions under homeostatic conditions and during activation and is reduced in a proportion of HSCs from both mice and human donors as they age. A further detailed understanding of these quality control mechanisms will contribute to the creation of new therapeutics to delay hematopoietic aging.

The unique configuration and the regulation of protein homeostasis or proteostasis networks (encompassing protein synthesis, folding, and trafficking, as well as degradation pathways) [419] are critical for long-term HSC maintenance and functions including their longevity, self-renewal, and hematopoietic regenerative potential [387]. A reduction in protein synthesis accompanies HSC quiescence [387]. Notable in vivo studies in mice have demonstrated that young adult bone marrow HSCs synthesize significantly lower amounts of proteins than lineage-restricted HPCs such as CMPs, GMPs, and MEPs [387,420] and do so independently of HSC size and cell cycle status and their content of total and ribosomal RNAs [331]. One consequence of low protein synthesis in HSCs is their increased ferroptosis, or iron-dependent programmed necrotic cell death [421,422]. The unique configuration of the long-lived HSC proteostasis network can also limit the abundance of misfolded and unfolded proteins and the accumulation of cytotoxic aggregated proteins and can maintain 3D protein conformation and in so doing limit proteotoxic stress and preserve HSC self-renewal potential [331,423,424,425,426]. While most cells rely on the proteosome to degrade misfolded proteins, HSCs, which possess low proteosome activity [425], rely on the preferential trafficking of misfolded proteins to aggresomes (defined as “*perinuclear inclusion bodies in which misfolded and aggregated proteins are concentrated and sequestered in a cage of intermediate filaments*”) and then on aggrephagy, a selective form of autophagy, as their main degradation pathway for protein aggregates [423]. However, the integrity of the proteostasis network and of the proteome declines in response to the stressors (e.g., inflammatory and oxidative stress) that accompany aging [379,427]. A severe loss of aggresomes, diminished autophagic flux, and increased proteosome activity are associated with HSC aging and together limit protein aggregate removal [423,424,425,426,427]. Molecular chaperones and cochaperones assist in ensuring correct protein folding in long-lived HSCs [418] and these may be compromised with aging. Cytophilin A (peptidyl-propyl isomerase A), which is associated with intrinsically disordered protein synthesis, is an example of an important HSPC chaperone with significantly reduced expression in aged HSCs leading to enhanced HSC aging and a decline in HSC quality [428]. While a detailed description of proteostasis is beyond the scope of this review, these examples serve to illustrate the importance of proteostasis to homeostatic hematopoiesis and its dysregulation with aging, as well as the need to further decipher the quality control mechanisms that exist in and preserve the functions of young adult long-lived HSCs. In particular, a greater understanding of human HSC dynamics and how they change over an individual lifespan is essential for creating new therapies that will improve blood regeneration and immune responses and address the age-related challenges [429] that affect the human hematopoietic system.

## 4. Conclusions

Major strides have been made in the therapeutic use of human HSPCs, particularly with regard to clinical transplantation. This has been aided by concurrent single-cell technological advances aimed not only at identifying and characterizing HSCs and their progeny at different stages of ontogeny but also at deciphering lineage relationships, stem and progenitor cell biases, and the mechanisms by which HSCs survive, remain dormant, exit quiescence, self-renew, or differentiate in specific niches or microenvironments. Despite this, our understanding of hematopoiesis has predominantly arisen from vast investigations using murine models. More recently, there have been renewed attempts to define human hematopoiesis in more detail. However, these efforts have faced major challenges as outlined in this review, but in part because human hematopoiesis does not necessarily recapitulate murine hematopoiesis. Furthermore, research into human hematopoiesis has been significantly hindered by limited access to human hematopoietic tissues and a dearth of robust methods with which to study the functions and lineage trajectories of human HSCs and their progeny in vivo. As an example, many studies use circulating HSPCs, such as those sourced from UCB at birth and from unperturbed or mobilized PB, to analyze post-natal human hematopoiesis, principally because of their relative ease of access. Yet, the bone marrow is recognized as the primary site for steady state hematopoiesis during human adult life, and the microenvironments to which human HSPCs are exposed in the circulation at different stages of ontogeny or after treatments with different mobilizing agents differ significantly from those found in healthy unperturbed human bone marrow. Additionally, as discussed in this review, the possibility has been raised that murine hematopoiesis may be differentially regulated at different bone marrow sites across the skeleton, especially in response to different stressors. Whether different bone marrow anatomical sites contribute equally to human hematopoiesis under steady state or perturbed conditions, such as sepsis, hemorrhage, or during HCTs, is not fully understood. Another major challenge is to develop methods to distinguish human homeostatic hematopoiesis from stress-induced or emergency hematopoiesis. This has been made even more difficult because functional in vivo analyses of human HSCs have generally relied on transplantation of ex vivo manipulated HSCs either into human recipients during clinical treatments for hematological disorders or into surrogate immunodeficient murine models, with the hematological response to transplantation thought to restrict HSC fate decisions and more likely reflect stress hematopoiesis as opposed to steady state hematopoiesis. The use of naturally occurring barcodes (e.g., mitochondrial mutations and epigenetic marks) provides promising approaches to tracing the fate of human HSCs in vivo under homeostatic and perturbed conditions. Further advances in lineage tracing and improved technological approaches will undoubtedly be developed to enhance our knowledge of human hematopoiesis in health and disease and to create new therapies and therapeutics for hematological and immune disorders that accompany aging.
